# Transfer of human α-synuclein from the olfactory bulb to interconnected brain regions in mice

**DOI:** 10.1007/s00401-013-1160-3

**Published:** 2013-08-08

**Authors:** Nolwen L. Rey, Geraldine H. Petit, Luc Bousset, Ronald Melki, Patrik Brundin

**Affiliations:** 1 Neuronal Survival Unit, BMC B11, Department of Experimental Medical Science, Wallenberg Neuroscience Center, Lund University, Sölvegatan 19, 221 84 Lund, Sweden; 2 Laboratoire d′Enzymologie et de Biochimie Structurale, UPR 3082 CNRS, Bâtiment 34, Avenue de la Terrasse, 91190 Gif-sur-Yvette, France; 3 Center for Neurodegenerative Science, Van Andel Institute, 333 Bostwick Avenue N.E., Grand Rapids, MI 49503 USA

**Keywords:** α-Synuclein, Olfactory pathway, Olfactory bulb, Transfer, Microglia, Parkinson’s disease

## Abstract

α-Synuclein (α-syn) is a protein prevalent in neural tissue and known to undergo axonal transport. Intracellular α-syn aggregates are a hallmark of Parkinson’s disease (PD). Braak and collaborators have suggested that in people who are destined to eventually develop PD, α-syn aggregate pathology progresses following a stereotypic pattern, starting in the olfactory bulb (OB) and the gut. α-Synuclein aggregates are postulated to spread to interconnected brain regions over several years. Thus, propagation of the pathology via neural pathways can potentially explain how α-syn aggregates spread in PD. We have now studied if α-syn can transfer from the OB to other brain structures through neural connections, by injecting different molecular species of human α-syn (monomers, oligomers, fibrils) into the OB of wild-type mice. We found that non-fibrillar human α-syn is taken up very quickly by OB neurons. Within minutes to hours, it is also found in neurons in structures connected to the OB. Conversely, when we injected bovine serum albumin used as a control protein, we found that it does not diffuse beyond the OB, is rarely taken up by OB cells, and does not transfer to other structures. Taken together, our results show that OB cells readily take up α-syn, and that monomeric and oligomeric, but not fibrillar, forms of α-syn are rapidly transferred to interconnected structures within the timeframe we explored. Our results support the idea that α-syn can transfer along neural pathways and thereby contribute to the progression of the α-syn-related pathology.

**Electronic supplementary material:**

The online version of this article (doi:10.1007/s00401-013-1160-3) contains supplementary material, which is available to authorized users.

## Introduction

Parkinson’s disease (PD) is characterized by abnormal cytoplasmic inclusions, mainly composed of misfolded α-synuclein (α-syn) [27, 58] in neuronal soma (Lewy bodies) and neurites (Lewy neurites). α-Syn is a soluble protein, structured in α-helices when associated to membranes. Under pathological conditions it oligomerizes and aggregates into fibrils arranged in β-rich sheet structures [16, 17, 57]. Despite the current lack of evidence that aggregated/misfolded α-syn is infectious between individuals [35], it has been proposed that aggregated/misfolded α-syn can behave in a prion-like fashion within the nervous system of one individual. This means that aggregated/misfolded α-syn can induce the native endogenous α-syn to adopt an abnormal conformation within the same cell. The misfolded α-syn is then released into the extracellular space and is taken up by a neighboring cell. Once inside the new cell, the misfolded α-syn can seed further α-syn misfolding and aggregation. Thereby the protein acts as a template for misfolding that transmits the disease process from one cell to another. This intercellular transfer of misfolded α-syn is hypothesized to contribute to the progressive spread of Lewy pathology in the nervous system in PD and other synucleinopathies [1, 11, 22, 30]. Earlier post-mortem studies of Lewy pathology in PD brains had led to the proposal that it gradually spreads throughout the nervous system following a predictable anatomical pattern [6, 8]. Braak and co-workers [5] suggested that Lewy pathology first develops in the olfactory bulb (OB), the anterior olfactory nucleus (AON), and in the dorsal motor nucleus of the vagus nerve, and based on neural connections to regions affected in later disease stages, they proposed that the pathology spread along protracted unmyelinated axons to interconnected regions. We, and others, have suggested that this spreading results specifically from axonal transport of α-syn, followed by a cell-to-cell transfer of misfolded α-syn to neighboring neurons. Numerous in vitro and in vivo studies show that α-syn can transfer between cells and seed aggregation in the recipient cell [2, 18, 19, 25, 29, 45, 50, 62]. Notably, the slow and fast intra-axonal transport of α-syn has also been well described in vitro [36, 56, 59], and studies using neurons in microfluidic chambers demonstrate that fibrillar and oligomeric α-syn can be taken up from the medium and transported intra-axonally in both anterograde [25] and retrograde [61] directions. Finally, recent studies demonstrated that α-syn aggregates appear in widespread brain areas, 1–6 months after injection of preformed α-syn fibrils into the striatum or cortex of mice [43, 44]. This suggests axonal transport of an aggregation-prone α-syn, although it is not absolutely clear if the α-syn aggregates can transfer from one neuron to another and develop in regions that are not directly connected to the injection sites. Moreover, the ability of α-syn to spread from anatomical regions suggested to be affected early in PD to brain areas that are impacted later on has so far not been explored. With the background that Lewy pathology in olfactory structures and impairments in olfaction both appear early in PD [20, 21], we targeted the OB. For the first time, we stereotactically injected different molecular species (monomers, oligomers composed of soluble high molecular weight species, and fibrils) of recombinant human α-syn into the OB of normal mice. We then characterized in detail the transfer of α-syn to interconnected brain regions over 72 h. Our findings indicate that α-syn monomers and oligomers are most rapidly and readily transferred from the injection site to interconnected brain regions. We observed a particular pattern of spread that matches known neural connections in the brain, but we cannot conclude with certainty that this transfer occurred by classical axonal transport. Our results supports the notion that transport of α-syn oligomers between interconnected regions might be important in the spreading of α-syn pathology between brain regions in PD.

## Materials and methods

### Animals

We purchased C57Bl/6J 3-month-old female mice from Charles River Laboratories and housed them six per cage under a 12-h light/12-h dark cycle with access to food and water ad libitum. The housing of the animals and all procedures were in accordance with the international guidelines and were approved by the Malmö-Lund Ethical Committee for Animal Research.

### Synthesis of different molecular species of recombinant wild type human α-syn

We expressed recombinant S-tagged wild-type (WT) human α-syn in *Escherichia coli* strain BL21 (DE3) (Stratagene) and purified it as described previously [26]. We determined α-syn concentration by spectrophotometry using an extinction coefficient of 5,960/M/cm at 280 nm. Pure α-syn (0.5 mM) in 50 mM Tris–HCl, pH 7.5, 150 mM KCl was filtered through sterile 0.22 μm filters and stored at −80 °C.

We obtained α-syn oligomers by incubating soluble S-tagged WT α-syn in 50 mM Tris–HCl (pH 7.5, 150 mM KCl) at 4 °C for 7 days without shaking. We separated oligomeric α-syn from monomeric α-syn by size exclusion chromatography (Superose 6 HR10/30, GE Healthcare). To achieve fibril assembly, we incubated soluble S-tagged WT α-syn under continuous shaking in an Eppendorf Thermomixer set at 600 rotations/min and 37 °C. We assessed the nature of the oligomeric and fibrillar species using a Jeol 1400 transmission electron microscope (Jeol Ltd.) following adsorption of the samples onto carbon-coated 200-mesh grids and negative staining with 1 % uranyl acetate. The images were recorded with a Gatan Orius CCD camera (Gatan).

### Fluorescent labeling

Monomeric and oligomeric S-tagged α-syn assemblies in 50 mM Tris–HCl (pH 7.5, 150 mM KCl) were buffer exchanged using NAP10 desalting columns (GE Healthcare) to phosphate buffered saline (PBS) buffer. We performed α-syn labeling with ATTO-550 NHS fluorophore following the manufacturer instruction (ATTO-Tec Gmbh) using a protein:dye ratio of 1:2. We removed unreacted fluorophore using NAP10 desalting columns. We centrifuged α-syn fibrils at 16,000*g* for 10 min (min) and resuspended them twice in PBS and labeled them as described above. We removed unreacted fluorophore by centrifugation at 16,000*g* for 10 min and then resuspended the pelleted fibrils twice in PBS. In the rest of the article, we have denoted recombinant α-syn tagged with ATTO-550 and S-tag “tα-syn”. Lyophilized BSA was purchased form Sigma (ref A9418), dissolved in PBS at 5 mg/mL and labeled with ATTO-550 dye using a protein:dye ratio of 1:2 following the same procedure as described for soluble tα-syn. Similarly, we have abbreviated BSA tagged with ATTO-550 as “tBSA”.

As control solutions, we will use unbound ATTO-550 and Alexa-488 (unbound to proteins). To obtain them, the NHS groups of ATTO-550 and Alexa-488 NHS (5 mM) were reacted with Tris–HCl (pH 7.5, 200 mM) as a primary amine source at room temperature for 3 h. The solution was then diluted to 200 μM in PBS and stored them at −80 °C.

### Stereotactic injections

We anesthetized 105 mice (3-month-old) with isoflurane/oxygen/nitrogen mixture, and stereotactically injected tα-syn or tBSA into the OB, or other intracranial sites (control injections) as specified below. We performed the stereotactic injections using a glass capillary attached to a 10 μl Hamilton microsyringe, and injected 0.8 μl of protein or fluorophore solutions unilaterally in the OB (coordinates: AP: +5.4 mm, *L*: −0.75 mm, DV: −1 mm relative to bregma and dural surface).

**Fig. 1:**
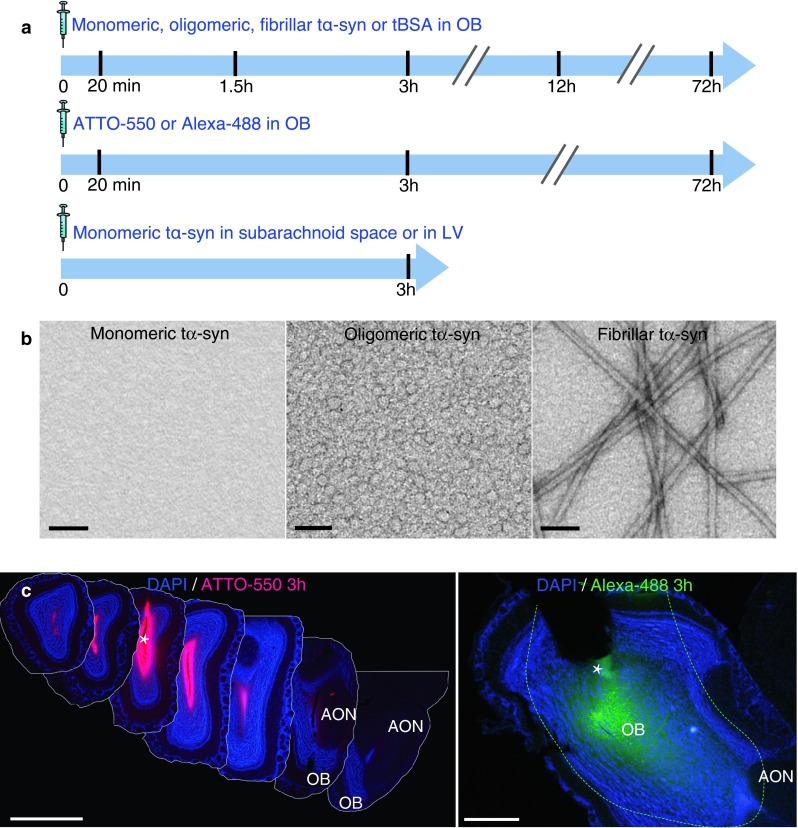
Experimental design and controls of diffusion. **a** Experimental design. In wild-type mice, we stereotactically injected α-synuclein (α-syn) into the olfactory bulb (OB), the lateral ventricle (LV) or at the level of subarachnoid space dorsal to the OB. Different molecular species of human recombinant α-syn tagged with ATTO-550 and S-tag (tα-syn) were injected: monomeric, oligomeric, or fibrillar tα-syn; or bovine serum albumin tagged with ATTO-550 (tBSA); or unbound ATTO-550 or Alexa-488 alone as control. We killed mice 20 min, 1.5, 3, 12 or 72 h after injection for histology. **b** Characterization of recombinant tα-syn. Negatively stained transmission electron micrographs of monomeric, oligomeric and fibrillar tα-syn. Oligomer samples are homogeneous, and do not contain any α-syn fibrils. *Scale bar* represent 100 nm. **c** Photographs of the injected region in the OB 3 h after injection of unbound ATTO-550 or Alexa-488 (dissolved in PBS) into the OB of mice. The images illustrate how far the injected solution diffused in the neural tissue. *Left panel* is a montage of coronal sections of a brain injected with unbound ATTO-550 (inter-section interval = 450 μm). Three hours after injection, ATTO-550 diffused from the injection site to lateral layers of the OB, and until the very anterior part of the AON, but not further. *Scale bar* 1 mm. *Right panel* is a low magnification picture of a sagittal section of a brain injected with Alexa-488. At 3 h after injection, Alexa-488 diffused from the injection site to the posterior part of the OB, the accessory olfactory bulb (AOB) and until the very anterior part of the anterior olfactory nucleus (AON), but not further. *Dashed line* represent the limit of Alexa-488 diffusion. *Scale bar* represent 500 μm. The *white asterisk* marks the injection site

The solutions we injected contained monomeric, oligomeric, fibrillar tα-syn, monomeric untagged α-syn or tBSA at a concentration of 1 mg/mL in sterile PBS. We perfused the mice 20 min, 1.5, 3, 12 or 72 h after injection and collected brains (4 mice per group; Fig. 1a; supplementary Table 1). To examine how far injected vehicle (containing no protein) can diffuse, we also injected mice with unbound ATTO-550 (9 mice) or unbound Alexa-488 in PBS (1 mg/mL, 6 mice) into the OB and perfused them at one of three time points (20 min, 3, 72 h).

To control for the possible spread of tα-syn within the brain via cerebrospinal fluid, we performed control injections of monomeric tα-syn in the subarachnoid space (on the top of the OB after breaking the dura mater; AP: +5.4 mm, *L*: −0.75 mm, DV: 0 mm relative to bregma and dural surface) or into the lateral ventricle (LV, AP: +0.74 mm, *L*: −0.6 mm, DV: −2.2 mm). We injected three animals per location and perfused these mice 3 h after injection. Following initial pilot experiments, we chose the volume of solution we injected so that it resulted in sufficient amounts of protein in the OB to allow the study of transport, but without giving rise to significant diffusion to other brain structures. Due to retrograde leakage of the injected protein along the injection capillary tract, we had to exclude one mouse in each of the following groups: monomeric tα-syn 1.5 h, oligomeric tα-syn 1.5 h, monomeric tα-syn 3 h and oligomeric tα-syn 3 h.

### Immunohistochemistry and analysis

#### Preparation of the tissue

At 20 min, 1.5, 3, 12 or 72 h after injection, we anesthetized mice with sodium pentobarbital and perfused them transcardially with 0.9 % saline followed by 4 % PFA in phosphate buffer. We dissected the brains, post-fixed them for 2 h in 4 % PFA and placed them in 30 % sucrose in phosphate buffer. We stored the brains at 4 °C until sectioning. We cut the entire brains into 30 μm free-floating coronal sections on a freezing microtome, and stored them in antifreeze solution at 4 °C until immunostaining. To control how far a protein-free solution could diffuse, we cut some brains injected with unbound Alexa-488 into 30 μm free-floating sagittal sections, and brains injected with unbound ATTO-550 into 30 μm free-floating coronal sections.

#### Diaminobenzidine (DAB) stainings

We stained coronal free-floating sections using primary antibodies anti-human α-syn syn211 (monoclonal raised in mouse, 1:500, Invitrogen) or anti-BSA (raised in rabbit, 1/1,000, Invitrogen) or anti-S-tag (1/1,000, Delta Biolab), and secondary antibodies biotinylated (Horse anti-mouse, 1/200, Vector lab; Donkey anti-rabbit 1/200, Abcam; Goat anti-rabbit, 1/200, Vector lab). For the detection of the antibody with DAB, we used a standard peroxidase-based method (Vectastain ABC kit, and DAB kit, Vector Laboratories) with nickel enhancement. S-tag staining was carried out on one mouse per group at 1.5 h to confirm our observations from syn211 staining. Sections were then counterstained by haematoxylin. We analyzed these sections by conventional light microscopy (Eclipse 80i microscope; Nikon).

#### Immunofluorescence staining

We stained coronal free-floating sections with primary antibodies anti-human α-syn syn211 (monoclonal raised in mouse, 1:500, Invitrogen), anti-Tuj1 (polyclonal raised in rabbit, 1/2,000, Covance), anti-Tuj1 (and monoclonal raised in mouse, 1/5,000, Covance), anti-BSA (raised in rabbit, 1/1,000, Invitrogen), anti-Iba1 (raised in rabbit, 1:500, Wako/Nordic labs) and with the appropriate secondary antibodies Alexa-488 anti-rabbit, Alexa-488 anti-mouse, Alexa-647 anti-mouse, Alexa-647 anti-rabbit (raised in goat, 1:400, Invitrogen). We analyzed these specimens with a confocal laser microscope Zeiss LSM 510, equipped with Ar and HeNe lasers.

#### Cell quantifications

We quantified the number of hu-α-syn positive cells in structures positive for hu-α-syn (diaminobenzidine staining) using a computer-assisted mapping and cell quantification program (Stereo Investigator, MBF Bioscience, Williston, USA) coupled to a Zeiss Imager M2 microscope (Carl Zeiss Microimaging, Göttingen, Germany). We analyzed all the positive structures in four animals per group, at 40× magnification at 1.5 h time point; the OB and the piriform cortex of four animals per group at 20 min time point and at every time point from 20 min to 3 h, respectively. In the OB, only mitral cells were analyzed. Every positive cell (exhibiting staining in the whole cell body, or punctate within the cell body) was counted in each structure on a determined number of sections per structure, spaced by 240 μm, and distributed along the rostrocaudal axis at equivalent locations in each animal. Then we calculated the total number of cells detected per structure. Kruskal–Wallis tests and post hoc Dunn’s multiple comparison analysis of the counting in the OB were performed on *n* = 4 per group, using Prism 6.0, Graphpad.

## Results

### Control of the composition and the stability of tα-syn preparations and experimental controls for stereotactic injections

Prior to injection, the monomeric, oligomeric and fibrillar states of recombinant α-syn were confirmed by transmission electron microscopy (Fig. 1a). To define the size of our oligomers, we performed size exclusion chromatography. We found that they are composed of soluble high molecular species, eluted at 2 MDa by this method (supplementary Fig. 1). It is worth noting that size exclusion chromatography does not allow an accurate measurement of the size of the protein since the mobility of the protein in the column depends also on its conformation and its charge [24]. Therefore, we cannot claim to know the precise size of our oligomers.

We also confirmed the stability of our different α-syn assemblies over 72 h period (supplementary Fig. 2). After a 72 h incubation at 37 °C, we found that the monomeric α-syn remains monomeric and soluble as shown by size exclusion chromatography (supplementary Fig. 2a, c) and sedimentation assay (supplementary Fig. 2e); oligomeric α-syn does not polymerize or disassemble (size exclusion chromatography, supplementary Fig. 2b, d), and fibrillar α-syn fibrils incubated in serum remain fibrillar (supplementary Fig. 2f).

We then controlled that our injections successfully targeted the rostrodorsal OB (supplementary Fig. 3a, b). To establish that any spreading of α-syn from the OB to other brain regions that we might observe was not due to non-specific diffusion or leakage from the injection site, we performed a series of important control experiments. Thus, to check that 0.8 μl of solution that we injected into the OB does not diffuse to other structures, we injected unbound ATTO-550 or Alexa-488 in PBS at the same coordinates used for tα-syn injection. We euthanized mice at different time points after injection (20 min, 3, and 72 h), dissected and cut brains sagittally or coronally to be able to monitor how far the injected solution diffused. We observed the maximum diffusion area at 3 h (Fig. 1c). At this time point, we observed (in sagittal and coronal sections) that the injected solution had diffused from the injection site in the OB to the anterior part of the AON. After 72 h, we only detected the control fluorophore in the central part of the OB (supplementary Fig. 3c).

### Neurons take up recombinant monomeric, oligomeric and fibrillar α-syn in less than 20 min after injection into the olfactory bulb

**Fig. 2:**
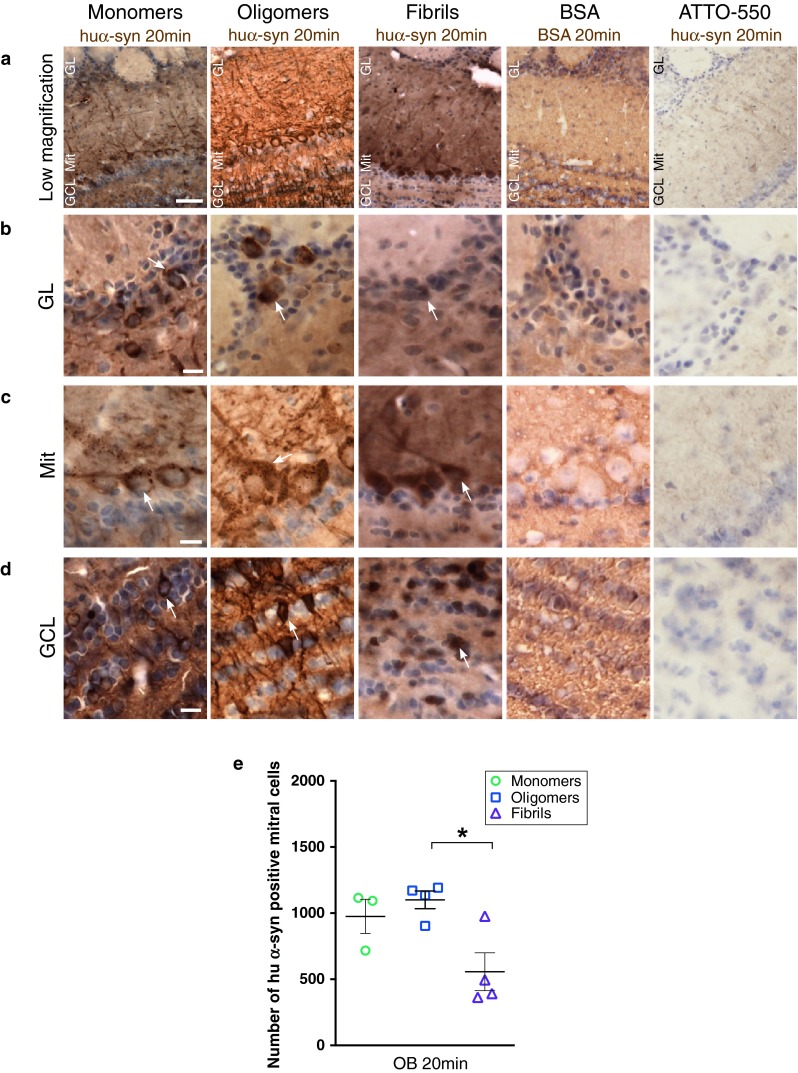
Tα-syn is taken up by OB cells less than 20 min after injection in the OB. Huα-syn- (stained with syn211 antibody) and BSA-staining in the OB, 20 min after injection of tα-syn, tBSA or ATTO-550 into the OB: **a** low magnification pictures of the ipsilateral OB (GL/Mit/GCL) (*scale bar* 50 μm), or high magnification pictures (*scale bar* 10 μm) of glomerular layer (GL) (**b**), mitral layer (Mit) (**c**), and granular cell layer (GCL) (**d**) of the ipsilateral OB. At 20 min after injection of monomeric, oligomeric and fibrillar tα-syn into the OB, we detected huα-syn-positive cells in various layers of the OB (GL, Mit, and GCL), but no BSA-positive cells in the OB injected with tBSA. A diffuse BSA-staining was observed in extracellular space. To control for specificity of our immunohistochemistry protocol, sections from groups that were injected with ATTO-550 into the OB were stained for huα-syn, and did not reveal any huα-syn-positive staining. A *white arrow* points examples of huα-syn-positive cells. **e** Uptake of injected α-syn at 20 min: number of huα-syn positive mitral cells in the ipsilateral OB at 20 min (*H* = 6.053, *P* < 0.04, post hoc test: monomers/fibrils *P* < 0.05). *Scatter plots* show data from individual mice

We sacrificed mice injected with tα-syn (monomers, oligomers or fibrils); tBSA or unbound ATTO-550 (as controls in the OB) 20 min after injection. Brain sections were stained with anti-human α-syn (huα-syn) antibody (syn211) or with anti-BSA antibody. In mice injected with monomeric, oligomeric and fibrillar tα-syn, we identified huα-syn-positive cells in different layers of the OB (Fig. 2a), i.e. the glomerular layer (GL, Fig. 2b), mitral cell layer (Mit, Fig. 2c), and in granule cell layer (GCL, Fig. 2d). The mitral cell layer contains the relay cells of the OB that project to central olfactory structures [49]. In contrast, no obvious BSA-positive signal in DAB was detected in OB cells (Fig. 2a–d). Instead, a diffuse signal in the extracellular space was seen. We also used the syn211 antibody to stain sections from brains injected with unbound ATTO-550. As expected, mice injected with only unbound ATTO-550 did not stain with the syn211 antibody and thus were negative for huα-syn (Fig. 2a–d; supplementary Table 2). Quantification, in DAB stained sections, revealed similar numbers of huα-syn positive mitral/tufted cells in the OB in groups injected with oligomers and monomers. The number of huα-syn positive mitral/tufted cells was, however, significantly lower in the group injected with fibrils (Fig. 2e).

**Fig. 3:**
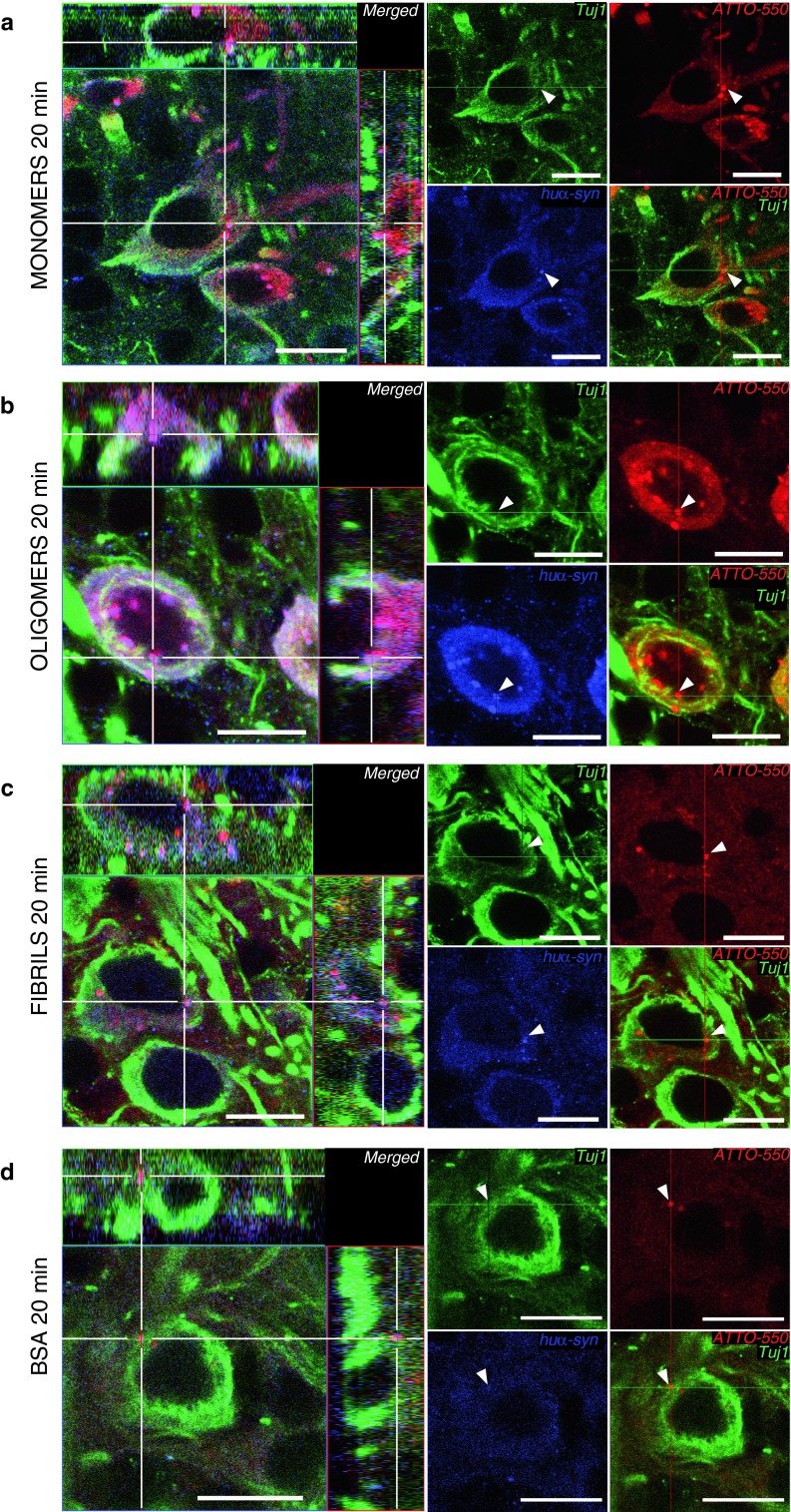
Tα-syn is localized within the somata of mitral cells in the OB 20 min after injection. We stained brain sections by immunofluorescence for Tuj1 (*green*), a neuronal marker, and for huα-syn or BSA. Tα-syn was identified both by its ATTO-550 fluorescent tag (*red*) and by the huα-syn (syn211 antibody) staining (*blue*). Similarly, in groups injected with BSA, the BSA was detected both by its ATTO-550 tag (*red*) and by BSA staining (*blue*). Confocal three-dimensional reconstructions (*large panels*) show ATTO-550 signal (*red*) colocalized with huα-syn staining (*blue*) within mitral cells (tuj1, *green*) in the OB of mice injected with monomers **(a)**, oligomers **(b)** and fibrils **(c)**, indicating that these cells contain huα-syn. We also occasionally detected BSA-positive punctae within Tuj1-positive mitral cells in mice injected with BSA **(d)**. *Smaller panels* are confocal plans showing tuj1 (*green*), ATTO-550 (*red*), huα-syn or BSA (*blue*) staining, and merged pictures of *green* and *red* channels. *Scale bars* represent 10 μm in every panel. *White arrowheads* point to tα-syn punctae

We detected tα-syn both by its ATTO-550 fluorescent tag (in red), and by huα-syn (syn211) immunostaining (in blue) using confocal microscopy (Fig. 3a–c). Similarly, we detected injected tBSA by visualizing its’ ATTO-550 fluorescent tag (in red), and by BSA immunostaining (in blue) (Fig. 3d). We detected neuronal cells by staining with a Tuj1 antibody (in green). In the mice injected with huα-syn monomers, oligomers or fibrils, we found that some Tuj1-immunoreactive mitral/tufted cells (Fig. 3a–c), granule cells, and periglomerular cells (data not shown), exhibited both ATTO-550 fluorescence and syn211-immunoreactivity confirming that they had taken up huα-syn. In mice in which we had injected monomers or oligomers into the OB, the syn211/ATTO-550 signal appeared both as a diffuse staining and as punctae within the cell bodies and processes of Tuj1-positive cells (Fig. 3a, b). In mice injected with fibrillar tα-syn, Tuj1-positive cells contained huα-syn rarely (Fig. 3c), consistently with what we observed in our quantifications (Fig. 2e). In mice injected with tBSA, very rare Tuj1-positive cell contained BSA-positive punctae (Fig. 3d).

These data demonstrate that mitral/tufted cells, periglomerular cells as well as granular cells readily take up monomeric and oligomeric tα-syn. Fibrillar tα-syn is also taken up, but to a much lesser extent within the time frame of the experiment. The high degree of neuronal uptake of injected tα-syn seems to be specific, as our control protein BSA was rarely taken up by neurons (Figs. 2a–d, 3d).

### Monomeric and oligomeric tα-syn appears in interconnected brain regions soon after injection into the olfactory bulb

#### 20 min post-injection

After injecting monomeric or oligomeric tα-syn into the OB, we found huα-syn-positive cells in the ipsilateral AON and occasionally (in one animal out of 4) in the ipsilateral frontal cortex (FC). After fibrillar tα-syn injections, we observed huα-syn-positive cells in the ipsilateral anterior AON only, in two mice out of four, event occurrence are summarized in supplementary Table 2, and immunohistochemistry results in supplementary Fig. 4. We did not observe BSA-positive signal in DAB in any non-OB brain region in control injections of tBSA.

#### 1.5 h post-injection

**Fig. 4:**
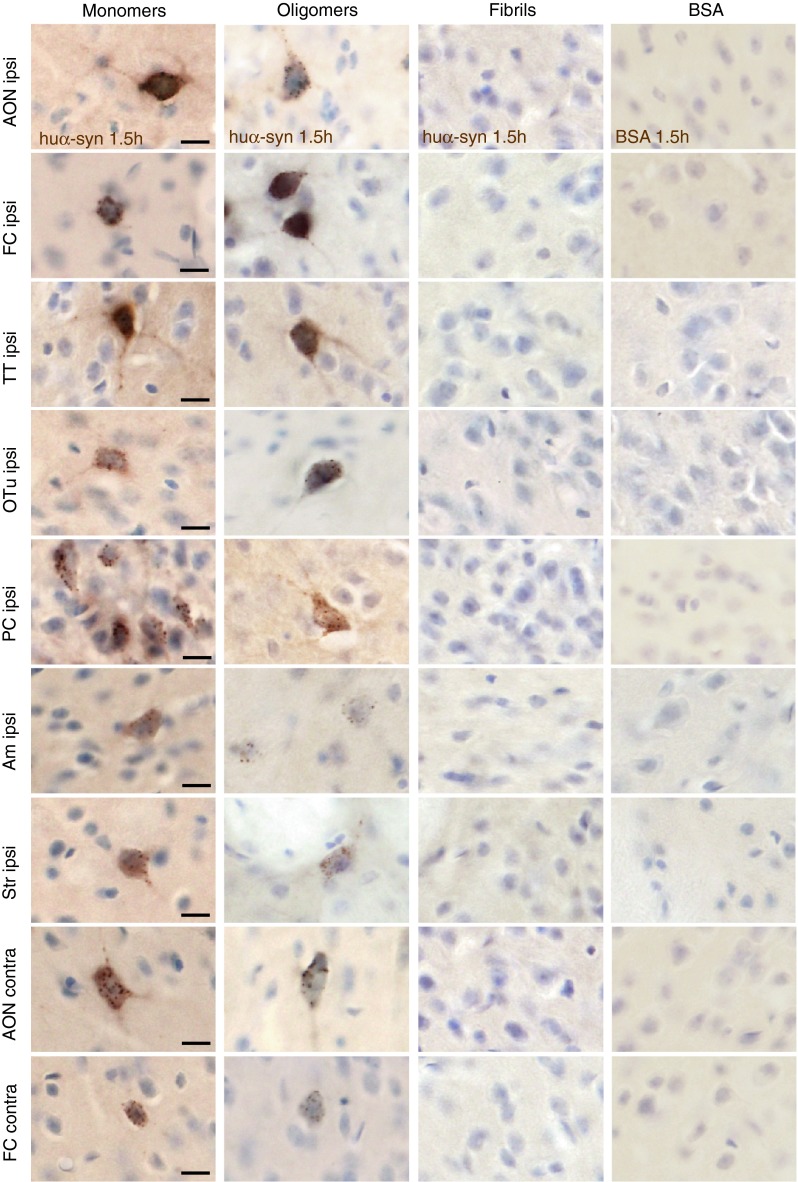
Transfer of tα-syn to other structures 1.5 h after injection into the OB. Images illustrating huα-syn or BSA staining at high magnification (*scale bar* 10 μm) in various brain areas. We detected huα-syn-positive cells in various brain structures 1.5 h after the injection of monomeric and oligomeric tα-syn into the OB. We observed huα-syn-positive cells in the ipsi- and contralateral anterior olfactory nucleus (ipsi/contra AON), in ipsi- and contralateral frontal cortex (ipsi/contra FC), in ipsilateral tenia tecta (ipsi TT), olfactory tubercle (ipsi Otu), piriform cortex (ipsi PC), amygdala (ipsi Am) and striatum (ipsi Str). On the contrary, we detected huα-syn-positive cells only in the ipsilateral OB when we injected fibrillar tα-syn. tBSA injected as a control protein into the OB, was not detected in the brain, except in the injected OB where we observed only a diffuse staining in extracellular space, but no obvious BSA-positive cell

**Fig. 5:**
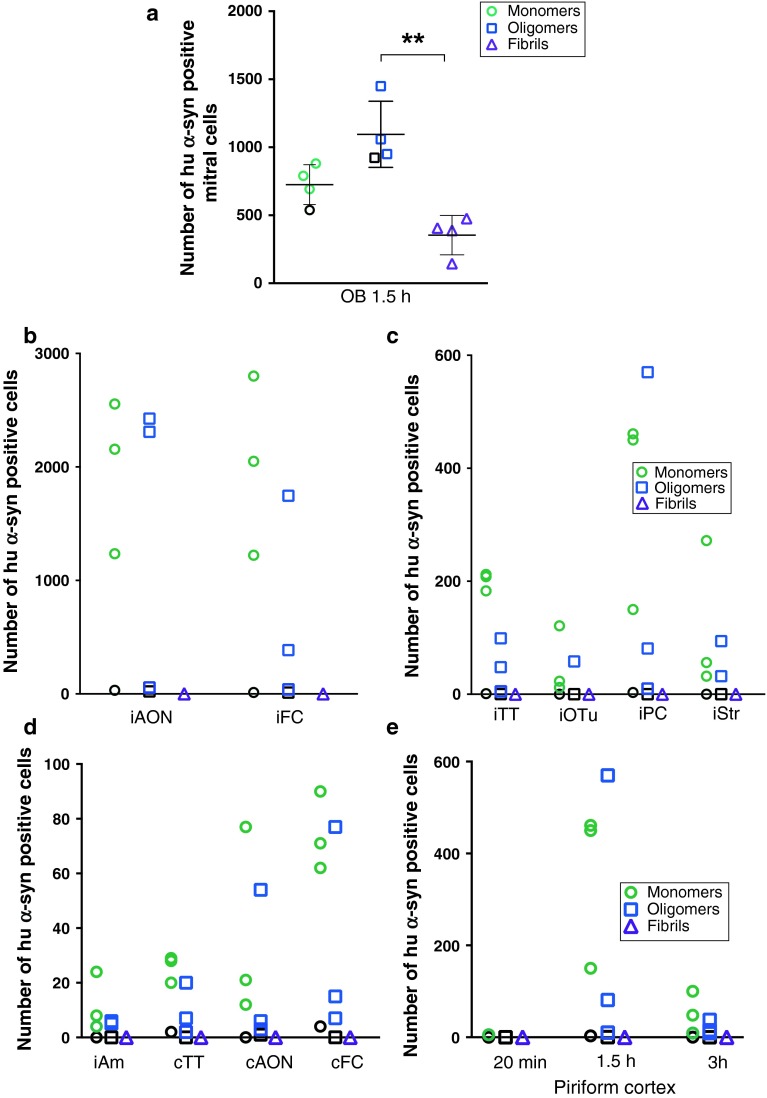
Quantifications of huα-syn-positive cells in different structures 1.5 h after injection into the OB**. a** Number of positive mitral cells in the ipsilateral OB at 90 min (*H* = 9.846, *P* < 0.01, post hoc test: monomers/fibrils *P* < 0.01). Positive cells in the ipsilateral anterior olfactory nucleus (iAON), ipsilateral frontal cortex (iFC) (**b**); ipsilateral tenia tecta (iTT), olfactory tubercle (iOTu), piriform cortex (iPC), and striatum (iStr) (**c**); ipsilateral amygdala (iAm) and contralateral tenia tecta (cTT), anterior olfactory nucleus (cAON) and frontal cortex (cFC) (**d**). **e** Time evolution of α-syn transfer and clearance in the ipsilateral piriform cortex. *Scatter plots* show data from individual mice. *Black symbols* represent animals that were excluded due technical issues during injection

At the 1.5 h time point, in mice injected with monomeric or oligomeric tα-syn in the OB, we detected huα-syn-positive cells in several additional brain ipsilateral-regions: namely the AON, the FC, the tenia tecta (TT), the olfactory tubercle (OTu), the piriform cortex (PC), the striatum and the amygdala. Huα-syn-positive cells were also present in the contralateral hemisphere, specifically in the FC and the AON (Fig. 4). The huα-syn-positive cells displayed both a diffuse perikaryal staining as well as a punctae staining pattern. Importantly, in ipsilateral structures located distant from the OB, e.g. as the striatum and the amygdala, we detected a few huα-syn-positive cells (a few cells per region) (Fig. 4). Brains injected with fibrillar tα-syn presented huα-syn-positive cells in the ipsilateral OB only (Fig. 4; supplementary Fig. 5a), suggesting that the fibrillar tα-syn was not transferred to any other brain region within 1.5 h. In brains given control injections of tBSA, we found no BSA-positive cells either inside or outside the OB. We only observed a diffuse extracellular staining in the injected OB and in the anterior part of the AON. In supplementary Table 3, we present details of the distribution of huα-syn- and BSA-positive staining in each group at 1.5 h. The quantification of huα-syn positive cells in groups injected with monomers and oligomers showed that the cells in anterior brain regions frequently exhibited huα-syn transfer. By contrast, in brain regions located further away from the OB (e.g. amygdala) the rate of huα-syn transfer was low (Fig. 5a–d). Schematic drawings of coronal brain sections in supplementary Fig. 6a–c depict the overall load of huα-syn-positive cells in the different brain regions.

Importantly, to confirm our data from huα-syn staining at 1.5 h time point, we also performed a staining directed against the S-tag of tα-syn. In each group of mice, we observed S-tag-positive cells in the same structures as with huα-syn staining. Thus the results obtained from S-tag detection are identical to the one from huα-syn staining (supplementary Fig. 7). Moreover, we confirm that the uptake by neurons and the transfer of huα-syn are not due to presence of the tags on huα-syn: injections of untagged monomeric huα-syn led the same distribution pattern as injections of tagged protein (supplementary Fig. 8; supplementary Table 3).

#### 3 h post-injection

**Fig. 6:**
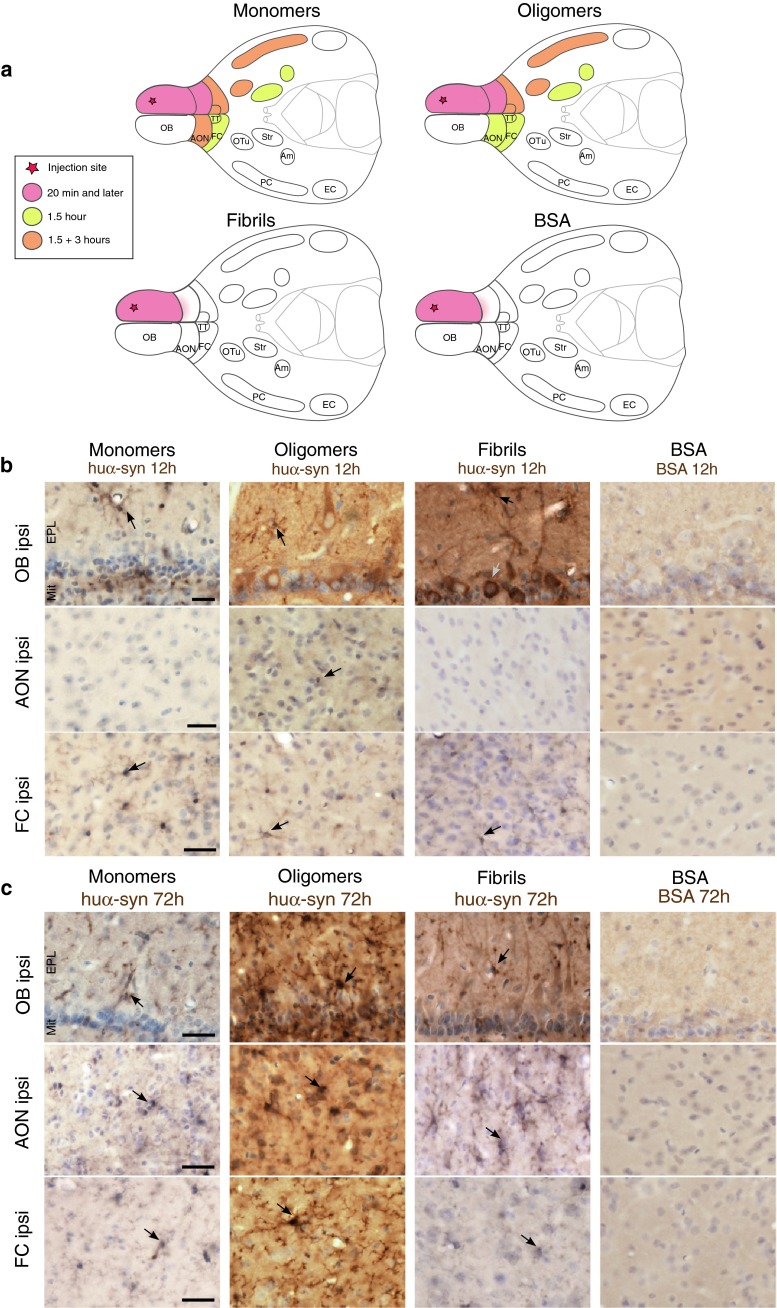
Temporal evolution of tα-syn presence in the brain after injection in the OB. **a** *Drawings* representing schematic horizontal sections of the brain with the different regions exhibiting huα-syn-positive cells or a diffuse BSA-staining at different time points after protein injection in the OB. **b** BSA and huα-syn staining 12 h after injection in the OB. We detected huα-syn-positive cells in the ipsilateral OB, and FC after injection of monomeric, oligomeric and fibrillar tα-syn, as well as in the ipsilateral AON after injection of oligomers. On the contrary, we did not find BSA-positive cells in the brain 12 h after tBSA injection in the OB. Huα-syn-positive cells appeared to be either mitral/tufted cells, located in the external plexiform (EPL) and mitral cell (Mit) layers of the OB (in groups injected with oligomers or fibrils, *grey arrows*), or displayed the morphology of microglial cells (in groups injected with monomers, oligomers or fibrils; *black arrows*). *Scale bar* represent 25 μm. **c** Huα-syn- and BSA-staining 72 h after injection in the OB. Huα-syn-positive cells were detected in the ipsilateral OB, AON and FC after injection of monomers, oligomers and fibrils 72 h after injection, and those cells exhibit a microglia-like morphology. On the contrary, no BSA-positive cell was found in the brain 72 h after tBSA injection in the OB. *Scale bar* represents 25 μm

3 h after injection of tα-syn monomers and oligomers into the OB, we still detected huα-syn-positive cells in some of the structures mentioned above (the ipsilateral AON, FC, TT, OTu, PC, and in the contralateral AON for monomers only). However, in contrast to the 1.5 h time point, we no longer found any huα-syn-positive cells in the ipsilateral amygdala, striatum and in contralateral FC (supplementary Fig. 9a; supplementary Table 4). In mice injected with fibrillar tα-syn, we still detect huα-syn-positive cells in the OB only. By contrast, mice receiving control injections of tBSA exhibited only diffuse BSA staining in ipsilateral OB and in the very anterior part of ipsilateral AON, and we observed no positively stained cell bodies in any brain region (data not shown). In Fig. 6a, we present a schematic drawing summarizing the progression of protein spread in the brain within the time frames we examined. Quantification of the number of positive cells in the piriform cortex show that the highest rate of transfer of monomeric and oligomeric tα-syn occurred within 1.5 h, followed by a strong decrease in the number of positive cells after 3 h (Fig. 5e). In supplementary Fig. 6a–c, we present schematic drawings of coronal brain sections showing the distribution of huα-syn-positive cells throughout the brain.

To control that the tα-syn we detected in brain regions far from the OB was not likely to be due to diffusion of the injected solution via the cerebrospinal fluid, we injected the same volume (0.8 μl) of monomeric tα-syn in the lateral ventricle (LV), or on the top of the OB, into the subarachnoid space. We killed mice 3 h after injection, and performed huα-syn immunostaining. We found huα-syn-positive cells around the injection site in the overlying cortex when the solution was injected into the LV. None of the brain structures mentioned above exhibited huα-syn-positive cells (AON, FC, PC, striatum, amygdala shown in supplementary Fig. 9b; supplementary Table 5). When we injected tα-syn on the top of the OB (in the subarachnoid space, we observed huα-syn-positive staining 3 h later only on the superficial part of the olfactory nerve at the level of injection. Thus we found no huα-syn-positive cells in the brain (negative staining in the OB, AON, FC, PC, striatum, amygdala shown in supplementary Fig. 9b; supplementary Table 5). These controls strongly suggest that tα-syn present in cells far from the OB is not the result of a diffusion of injected tα-syn through the cerebrospinal fluid. Taken together, our results suggest that after injection into the OB, monomeric and oligomeric tα-syn is taken up by neurons locally and rapidly spreads from the OB to other brain structures.

### More than 12 h after injection into the olfactory bulb, α-synuclein is present in neurons and microglia locally, as well as in microglia in interconnected brain regions

We also investigated the localization of tα-syn at two later time points, 12 and 72 h post-injection.

#### 12 h post-injection

After injections of monomers and fibrils into the OB, we detected tα-syn in cells with a microglia-like shape in the ipsilateral OB and FC; and occasionally in the AON, in one to two animals out of four. After injection of oligomers, we observed huα-syn-positive cells with microglia-like shape in the ipsilateral OB, FC and AON (Fig. 6b; supplementary Table 6). Interestingly, in the mice that received injections of oligomers or fibrils into the OB, we also identified huα-syn-positive mitral and tufted cells, based on their shape and precise localization using DAB staining (Fig. 6b, representative sketches in supplementary Fig. 6b, c).

**Fig. 7:**
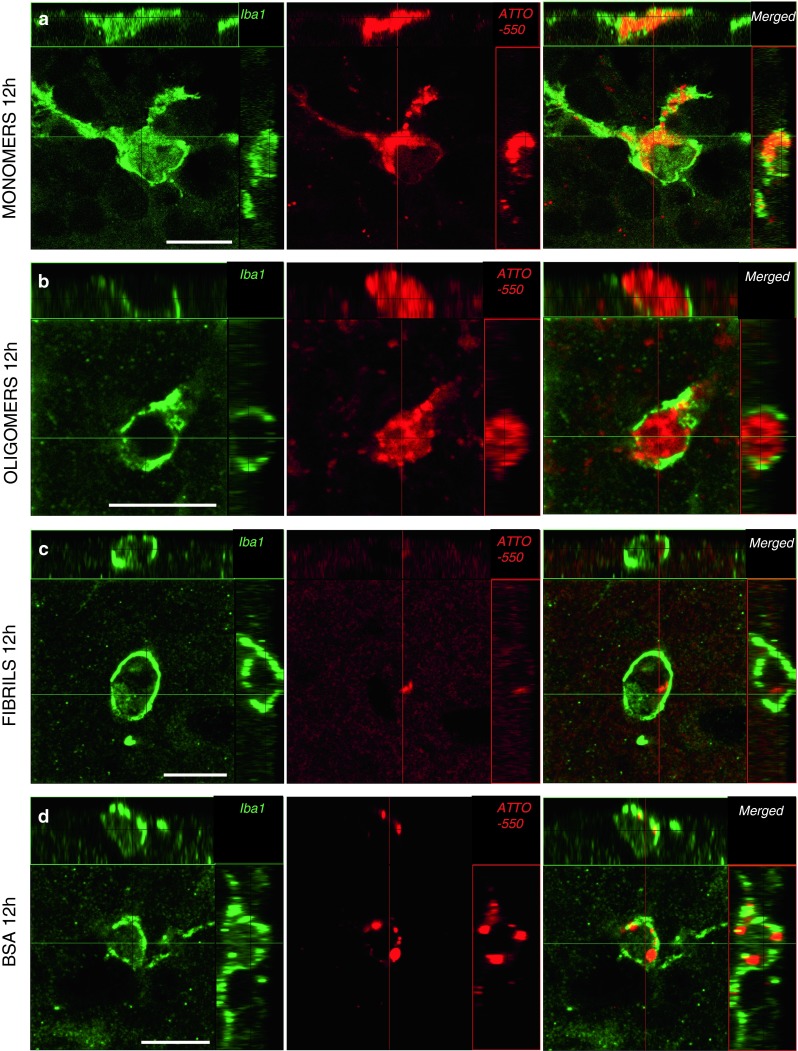
Tα-syn is localized within microglia 12 h after injection into the OB. Sections were stained by immunofluorescence for Iba1 (*green*), a microglial marker. Tα-syn and tBSA were identified by their ATTO-550 fluorescent tag (*red*). Confocal three-dimensional reconstructions show ATTO-550 signal (*red*) colocalized with Iba1 (*green*) within microglia in the OB of mice injected with monomers **(a)**, oligomers **(b)** and fibrils **(c)**, indicating that these microglial cells contain huα-syn. We also detected ATTO-550 signal in Iba1-positive cells in mice injected with the control protein tBSA **(d)**. *Scale bars* represent 10 μm in each panel

We confirmed the presence of tα-syn in the perikarya of microglia in the OB by immunofluorescent staining for Iba1 after injection of monomeric (Fig. 7a), oligomeric (Fig. 7b) and fibrillar tα-syn (Fig. 7c). Rarely we found microglial cells containing BSA-positive punctae (detected with its ATTO-550 tag) (Fig. 7d) 12 h after injection of tBSA.

#### 72 h post-injection

**Fig. 8:**
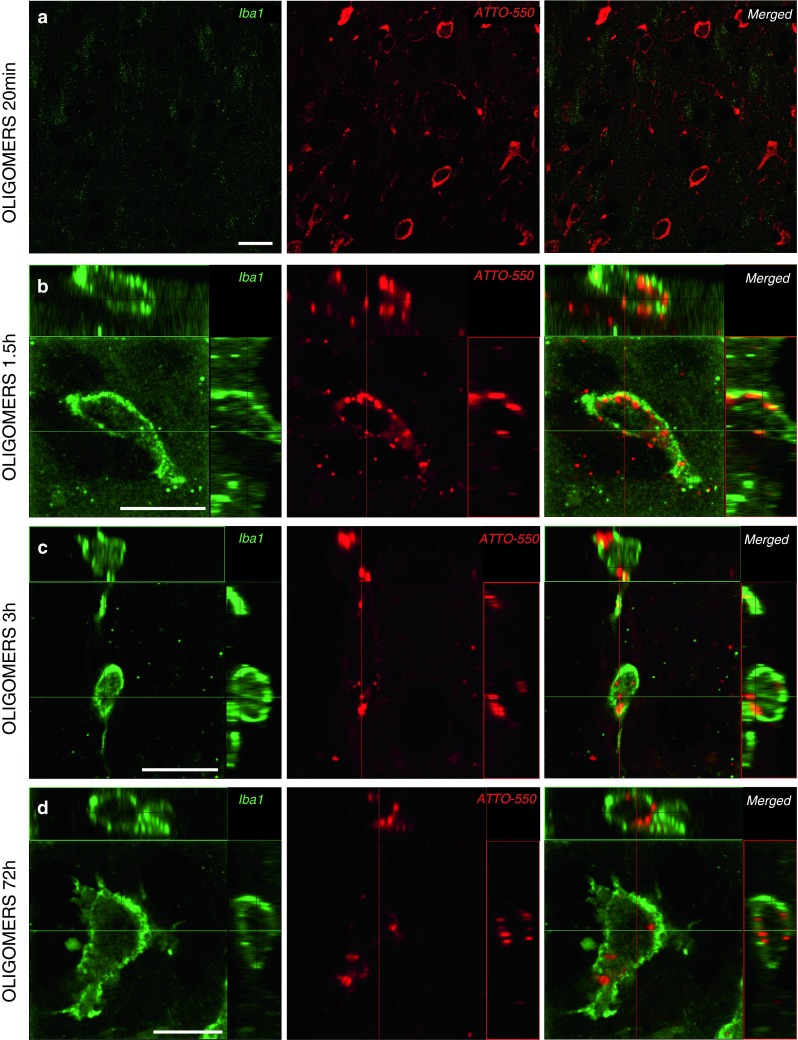
Tα-syn is localized within microglia already 1.5 h after injection into the OB. Tα-syn was identified by its ATTO-550 fluorescent tag (*red*), and microglial cells by Iba1 staining (*green*) by confocal microscopy. At 20 min after injection of oligomers, no Iba1-positive cell containing ATTO-550 signal was detected **(a)**. After 1.5 h, confocal three-dimensional reconstructions show ATTO-550 signal (*red*) colocalized with Iba1 (*green*) also within microglia in the OB of mice injected with oligomers at 1.5 h **(b)**, 3 h **(c)** and 72 h **(d)** timepoints, indicating that these cells contain huα-syn. *Scale bars* represent 10 μm in each panel

At 72 h after injection of tα-syn monomers, oligomers and fibrils, we observed heavily huα-syn-positive microglia cells in the ipsilateral OB and FC, as well as the ipsilateral AON (Fig. 6c; supplementary Table 7). Again, we confirmed the presence of tα-syn (detected using ATTO-550 tag) in the perikarya of microglia in the OB by immunofluorescence after injection of tα-syn (Fig. 8d).

#### Recruitment of microglia in the OB 1.5 h after injection of tα-syn

The first microglial cells appear in the OB around the injection site 1.5 h after injection of oligomeric tα-syn. While we could not detect Iba1-positive cells at 20 min (Fig. 8a), we observed tα-syn in the perikarya of microglia in the OB at later timepoints, from 1.5 to 72 h after injection (Fig. 8b–d).

## Discussion

Here we demonstrate that huα-syn is taken up by neurons in the OB and can also be rapidly transferred to interconnected brain regions. Specifically, local OB interneurons as well as mitral/tufted cells (relay cells of the OB that project to further olfactory structures) readily take up monomeric and oligomeric huα-syn. The same neurons also take up fibrillar huα-syn, but to a much lesser extent. The OB cells also take up tBSA, but to a lesser degree, suggesting that monomeric and oligomeric huα-syn are readily taken up and subject to transfer to other brain regions. Consistent with previous observations made in vitro [41], α-syn low molecular species are rapidly taken up resulting in some OB cells being positive for huα-syn already 20 min after injection.

### Non-specific/passive diffusion probably plays a minor role in spreading of α-synuclein in the brain

We performed different control injections and the results showed that it is highly unlikely that the injected material diffuses away from the OB via the brain parenchyma or the cerebrospinal fluid and thereby contributes to the spread of injected huα-syn to other brain regions. Therefore the huα-syn we observed in neurons in brain regions distant from the OB is more likely to have been transported intracellularly via neural projections, considering the pattern of transfer to structures interconnected with the OB.

Regarding the different molecular species of huα-syn we used, we observed clear differences in the transfer of fibrils and monomers/oligomers. We found that monomers and oligomers are taken up by neurons in the OB and are rapidly transported to other brain regions. In contrast, fibrils are taken up by fewer neurons in the OB and they are not transferred at detectable levels to other brain regions within 72 h.

In vitro, the internalization of fibrils by neurons is slower than that of soluble α-syn and can take several hours [41]. This relatively slow uptake of fibrils, combined with a rapid and efficient clearance of extracellular huα-syn by microglia, could limit the load of fibrils that are taken up by neurons.

### Alternative spreading mechanisms to intra-axonal transport

Our data suggest that the injected huα-syn does not spread via the cerebrospinal fluid. We cannot, however, rule out the involvement of other mechanisms in huα-syn spread from the OB. Entry of huα-syn into the blood circulation by physically damaged vessels at the injection site, and transport of huα-syn via blood vessels is one such option. In the mouse, huα-syn injected intravenously has a half-life of about 1 h [37]. However, there is no spread to structures that are not connected via neural pathways to the OB, suggesting that vascular transfer is not involved. Another possibility is huα-syn is engulfed by microglial cells [4, 64] that then migrate from one brain region to another. If the huα-syn is not successfully degraded by the microglia it is conceivable that it is released into the extracellular space and then is taken up by adjacent cells. Once again, however, then one would not expect to find huα-syn positive neurons only in regions directly connected via neural pathways to the OB. Finally, two other alternative mechanisms to microtubule-dependent intracellular transport that could explain the specific pattern in interconnected regions need to be considered. Either simple diffusion of huα-syn within the neuronal cytoplasm could play a role, or myosin-actin-dependent transport [9], that is reported to support transport (of organelles) within axons and dendrites, is operative. For both these mechanisms, however, the speed of transport is too low to explain our observations. Furthermore, short actin filaments are more likely to contribute to short-distance intra-neuronal transport as opposed to long-range axonal transport [9].

### Direction of α-synuclein transfer

Several in vitro and in vivo studies strongly suggest that α-syn can be released by cultured cells or neurons in vivo and taken up from the culture medium or from extracellular compartment by neighboring cells [2, 19, 25, 29]. In addition, other in vitro studies have demonstrated that α-syn is transported within the axon of a recipient neuron in both anterograde and retrograde directions [25, 61]. Therefore, if the spread of huα-syn we observe is due to an axonal transport from the OB to interconnected region, transport along axons in both directions, combined with cell-to-cell transfer at synaptic sites, could explain why we observed huα-syn in brain regions outside the OB.

**Fig. 9:**
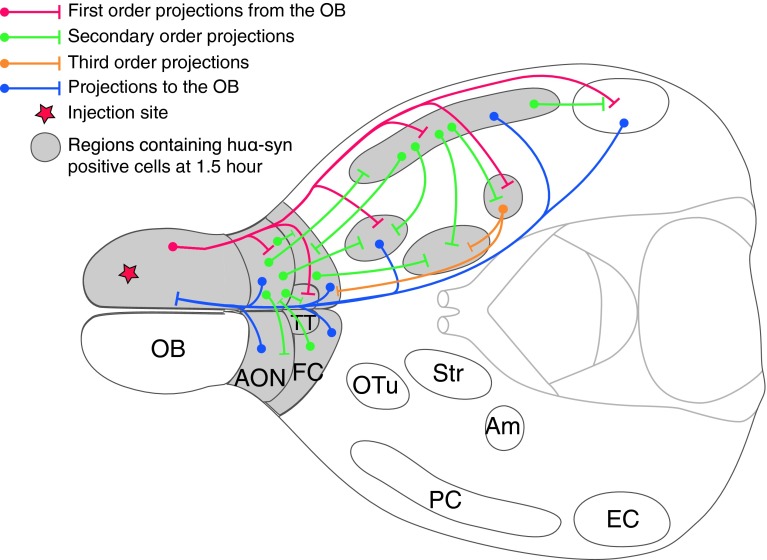
Schematic diagram summarizing both the olfactory neural network and regions where huα-syn-positive cells are detected after injection into the OB. This schematic horizontal section of the brain illustrate some of the important regions connected to the OB. *Red*, *green*, *yellow* and *blue* pathways schematically represent connections within the olfactory system. Circular endpoint of pathways represents the cell body of a relay neuron and a strait end represents the axonal terminal. *Structures filled with grey* represent regions that displayed huα-syn-positive cells 1.5 h after injection of monomeric and oligomeric tα-syn. All those regions are directly, or indirectly connected to the OB by centrifugal or centripetal projections

First, with this hypothesis in mind, let us consider that huα-syn undergoes anterograde axonal transport from the OB in our paradigm (see Fig. 9), a notion consistent with the dual-hit hypothesis [31]. Almost every structure where we detected huα-syn-positive neurons receives direct axonal projections from OB mitral/tufted cells (ipsilateral AON, TT, OTu, PC, amygdala; [12, 28, 33, 48]). Thus, it is possible that the injected huα-syn underwent anterograde transport from the OB to these structures within the axons of mitral/tufted cells, and then transferred from the axon termini to the target neurons. Regarding the brain structures that lack direct axonal projections from the OB but that still exhibited huα-syn-positive neurons (ipsilateral striatum, contralateral AON and both ipsilateral and contralateral FC), anterograde transport in two steps could explain the spread we observed. For example, the huα-syn might have been transported via the PC/amygdala to the striatum, followed by a cell-to-cell transfer to striatal neurons.

Second, still in the light of a possible intra-axonal transfer, huα-syn could also have been transported retrogradely from the OB to connected brain regions. The OB receives projections from almost all the structures where we detected huα-syn, namely the AON bilaterally [34, 54], FC [14], ipsilateral PC and OTu [47] and the ipsilateral TT [15, 52]. Thus, retrograde transport along axons to cell bodies located in these aforementioned regions (AON, FC, PC, OTu, TT) is plausible.

We also found labeled protein in two brain regions that do not project to the OB, i.e. the amygdala and striatum. The huα-syn in the amygdala could be the result of direct retrograde transport from the accessory OB along axons originating in the amygdala [53]. Alternatively, the huα-syn underwent retrograde transport from the OB to, e.g. the ipsilateral FC and transcended one synapse before undergoing further retrograde transport to the amygdala [40]. Thus, for all the structures cited above, both anterograde or/and retrograde transport(s) are conceivable. In some cases, the locations in which we found huα-syn require that the protein had transcended at least one synapse. The huα-syn we found in the striatum cannot be the result of retrograde transport due to the lack of direct (or indirect) projections from the striatum to the OB. Thus, if and intra-axonal transport to the striatum occurred, it can only be via anterograde transport with a transsynaptic step before reaching striatal neurons.

Finally, the entorhinal cortex, which receives direct projections from the OB, did not contain any huα-syn-positive cells. The transfer to the entorhinal cortex might require more than 1.5 h considering the long distance between the entorhinal cortex and the OB, and transferred protein may be cleared from the cells before the next (3 h) time point we studied. Another explanation could be that the concentration of transferred huα-syn might be below DAB staining threshold. Alternatively, huα-syn may be either taken up inefficiently by neurons from the entorhinal cortex.

### Speed of α-synuclein spread is compatible with intra-axonal transport

At 1.5 h after injection into the OB, we observed huα-syn in structures located at 4.8 mm maximum distance from the posterior limit of injected area in the OB (maximal speed 3.2 mm/h). At 20 min after injection, huα-syn reaches areas located less than 0.6 mm approximately caudal to the diffusion area in the OB (maximal speed 1.8 mm/h). From the perspective of axonal transport, this range of speeds is compatible with both retrograde (4–8 mm/h) and fast anterograde transport (2.1–16.6 mm/h) (reviewed in [10, 51, 55]. In fact, 15–26 % of slow component b particles and α-syn can be transported via fast axonal transport [36, 56], and thus might account for the presence of huα-syn in structures that are relatively distant from the OB.

### Rapid disappearance of injected α-synuclein from neurons

At 3 h after injection, no additional (compared to the 1.5 h time point) brain regions contained huα-syn. In some of the structures where the staining obtained 1.5 h after injection was very discrete, it had disappeared at the 3 h time point. At that time, no huα-syn was detectable in the amygdala, striatum and brain regions further downstream. At 12 h, but not at 72 h a few huα-syn-positive neurons were still present in OBs injected with oligomers and fibrils. Interestingly, we also detected huα-syn-positive microglia in the OB, AON and FC. These were rare at 1.5 and 3 h, but prevalent at 12 and 72 h. Taken together, these data support the idea that the huα-syn is gradually cleared from the neurons. It is likely that huα-syn is released to the extracellular space by neurons, or that some neurons containing huα-syn died, leading to the huα-syn (free or bound to cellular debris) being cleared by microglia [42].

### Relevance of α-synuclein transfer to the spread of Lewy pathology in patients

Regarding the spatial evolution of Lewy pathology in PD brains, Braak and co-workers [6, 8] proposed that synucleinopathy starts in the OB and AON, and/or in the dorsal motor nucleus of the vagus nerve; and then spreads to others structures along neural connections [6–8, 31, 32].

The first hints that such a progression of neuropathology is plausible are now coming from animal models of PD. For example, Ulusoy et al. [60] recently reported caudo-rostral spreading of α-syn in the medulla oblongata following injection of adeno-associated viral vector expressing huα-syn into the vagus nerve. Moreover, Luk et al. [43] recently demonstrated in mice that injections of pathologically misfolded α-syn into the striatum induce a PD-like pathology in different brain regions, along with motor dysfunction and dopaminergic nigral cell loss. The pathology took months to develop and was initiated by injecting huα-syn in the striatum, i.e. a brain structure that is not affected early in PD. Importantly, we investigated in a very short time-frame, the transfer of huα-syn itself, from the OB, which is thought to be one of the two regions affected first in PD, to other brain regions [6, 8]. We know now that huα-syn can be transferred from the OB of WT mice to interconnected regions, within a very short time frame and is cleared very efficiently, presumably by microglia. Although others have found that certain forms of preformed α-syn fibrils can trigger a PD-like pathology in mouse brain [43, 44, 46], we saw no Lewy-like bodies after injecting our huα-syn fibrils into the OB. Differences in the dose and the nature of the injected form of α-syn, e.g. monomers vs oligomers vs fibrils, as well as differences in the duration of the experiment, could explain why we did not observe the formation of Lewy-like bodies. Alternatively, the mouse OB might handle α-syn fibrils differently to the striatum. Interestingly, the role of α-syn oligomers in PD is also highly debated [38, 39], and some recent studies suggest that this form of α-syn is particularly toxic [19, 23, 63] and plays a key intermediary role in α-syn fibrillization [3]. A recent paper suggests that specifically α-syn oligomers, but not monomers and fibrils, disturb membrane trafficking when cultivated cells are exposed to them in the culture medium [13]. With this background, the high propensity of oligomers to spread between brain regions, which we observed in our model, may be important also in a disease context. We observed that monomeric and oligomeric α-syn can be transferred to interconnected regions. From that, we can speculate that a pathological seed of α-syn in the same range of size as our monomers–oligomers could also be transferred in an identical manner to interconnected regions. Moreover, if the α-syn escapes from proteostatic clearance, it can slowly recruit other proteins (including α-syn from the recipient cell) and thereby contribute to the spread of synucleinopathy within the brain.

## Conclusion

We have demonstrated for the first time that huα-syn injected into the OB is taken up by neurons and transferred to interconnected brain regions. This transfer is consistent with anterograde and/or retrograde axonal transport as well as cell-to-cell transfer. Our data support the concept that α-syn (main component of Lewy bodies) can spread from the OB, one of the first structures affected in PD according to Braak and collaborators, to interconnected brain regions.

## Electronic supplementary material

Below is the link to the electronic supplementary material.

Supplementary tables (PDF 350 kb)

Supplementary Figure 1 (PDF 87 kb)

Supplementary Figure 2 (PDF 786 kb)

Supplementary Figure 3 (PDF 2186 kb)

Supplementary Figure 4 (PDF 668 kb)

Supplementary Figure 5 (PDF 2070 kb)

Supplementary Figure 6 (PDF 3961 kb)

Supplementary Figure 7 (PDF 712 kb)

Supplementary Figure 8 (PDF 376 kb)

Supplementary Figure 9 (PDF 521 kb)
