# Supplementary material for: Transfer of human α-synuclein from the olfactory bulb to interconnected brain regions in mice
Source: Acta Neuropathol. 2013 Aug 8;126(4):555–73. doi: 10.1007/s00401-013-1160-3 (PMC3789892; doi:10.1007/s00401-013-1160-3)
Supplement: Supplementary file 1 — Supplementary tables (PDF 350 kb) [file 401_2013_1160_MOESM1_ESM.pdf]

Supplementary material

Transfer of human α-synuclein from the olfactory bulb to interconnected brain regions in mice

Nolwen L Rey, Geraldine H Petit, Luc Bousset, Ronald Melki and Patrik Brundin

Suppl table 1: Groups and number of animals

Injection into	Survival time	Tagged monomers	Untagged monomers	Tagged oligomers	Tagged fibrils	Tagged BSA	ATTO -550	Alexa -488
OB	20min	n=4		n=4	n=4	n=4	n=3	n=2
OB	1.5h	n=4	n=4	n=4	n=4	n=4		
OB	3h	n=4		n=4	n=4	n=4	n=3	n=2
OB	12h	n=4		n=4	n=4	n=4		
OB	72h	n=4		n=4	n=4	n=4	n=3	n=2
Subarach-noid space	3h	n=3						
LV	3h	n=3						

Supplementary table 1: Groups and number of animals. Numbers of mice in each experimental group. There are 26 different groups, based on injection site, the protein injected and the time between injection and perfusion (survival time). Lateral ventricle (LV), Subarachnoid space dorsal to the OB.

Supplementary table 2: Number of positive events per group. 20 min timepoint.

Groups	OB ipsi	AON ipsi	AON contra	FC ipsi	FC contra	TT Ipsi	OTu ipsi	PC ipsi	Am ipsi	Str ipsi
Monomers	4/4	3/4	0/4	1/4 (rare)	0/4	0/4	0/4	0/4	0/4	0/4
Oligomers	4/4	1/4	0/4	1/4 (rare)	0/4	0/4	0/4	0/4	0/4	0/4
Fibrils	4/4	2/4	0/4	0/4	0/4	0/4	0/4	0/4	0/4	0/4
BSA	4/4	2/4 ant AON	0/4	0/4	0/4	0/4	0/4	0/4	0/4	0/4
ATTO550	0/3	0/3	0/3	0/3	0/3	0/3	0/3	0/3	0/3	0/3

Supplementary table 3: Number of positive events per group. 1.5 h timepoint.

Groups	OB ipsi	AON ipsi	AON contra	FC ipsi	FC contra	TT Ipsi	OTu ipsi	PC ipsi	Am ipsi	Str ipsi
Monomers*	3/3	3/3	3/3	3/3	3/3	3/3	3/3	3/3	3/3	3/3
Oligomers*	3/3	3/3	3/3	3/3	3/3	3/3	1/3	3/3	2/3	2/3
Fibrils	4/4	0/4	0/4	0/4	0/4	0/4	0/4	0/4	0/4	0/4
BSA	4/4	2/4 ant AON	0/4	0/4	0/4	0/4	0/4	0/4	0/4	0/4
ATTO-550										
Untagged monomers	4/4	4/4	4/4	4/4	3/4	4/4	4/4	4/4	2/4	3/4

Supplementary table 4: Number of positive events per group. 3 h timepoint.

Groups	OB ipsi	AON ipsi	AON contra	FC ipsi	FC contra	TT Ipsi	OTu ipsi	PC ipsi	Am ipsi	Str ipsi
Monomers*	3/3	3/3	1/3	3/3	0/3	2/3	3/3	3/3	0/3	0/3
Oligomers*	3/3	3/3	0/3	2/3	0/3	2/3	1/3	3/3	0/3	0/3
Fibrils	4/4	0/4	0/4	0/4	0/4	0/4	0/4	0/4	0/4	0/4
BSA	4/4	2/4 ant AON	0/4	0/4	0/4	0/4	0/4	0/4	0/4	0/4
ATTO-550	0/3	0/3	0/3	0/3	0/3	0/3	0/3	0/3	0/3	0/3

Supplementary table 5: Number of positive events per group. Controls 3 h timepoint.

Monomers injected	Injection site	OB ipsi	AON ipsi	FC ipsi	TT ipsi	OTu ipsi	PC ipsi	Am ipsi	Str ipsi
In LV	Around inj site: 3/3	0/3	0/3	0/3	0/3	0/3	0/3	0/3	0/3
In subarac. space	OB under inj site: 3/3	0/3	0/3	0/3	0/3	0/3	0/3	0/3	0/3

Supplementary table 6: Number of positive events per group. 12 h timepoint.

Groups	OB ipsi	AON ipsi	AON contra	FC ipsi	FC contra	TT Ipsi	OTu ipsi	PC ipsi	Am ipsi	Str ipsi
Monomers	4/4	1/4	0/4	3/4	0/4	0/4	0/4	0/4	0/4	0/4
Oligomers	4/4	4/4	0/4	4/4	0/4	0/4	0/4	0/4	0/4	0/4
Fibrils	4/4	2/4	1/4	4/4	1/4	0/4	0/4	0/4	0/4	0/4
BSA	4/4	0/4	0/4	0/4	0/4	0/4	0/4	0/4	0/4	0/4
ATTO-550										

Supplementary table 7: Number of positive events per group. 72 h timepoint.

Groups	OB ipsi	AON ipsi	AON contra	FC ipsi	FC contra	TT Ipsi	OTu ipsi	PC ipsi	Am ipsi	Str ipsi
Monomers	4/4	3/4	0/4	3/4	0/4	0/4	0/4	0/4	0/4	0/4
Oligomers	4/4	4/4	0/4	4/4	0/4	0/4	0/4	0/4	0/4	0/4
Fibrils	4/4	3/4	1/4	4/4	0/4	0/4	0/4	0/4	0/4	0/4
BSA	0/4	0/4	0/4	0/4	0/4	0/4	0/4	0/4	0/4	0/4
ATTO-550	0/3	0/3	0/3	0/3	0/3	0/3	0/3	0/3	0/3	0/3

Supplementary table 2-7: Number of positive events per group.

Each fraction represents the proportion of mice exhibiting huα-syn-positive cells in the named structure (ipsi: ipsilateral; Contra: contralateral) after injections of tα-syn or ATTO-500, or untagged α-syn, or exhibiting a positive extracellular staining for BSA after tBSA injection, out of the total number of animals in each group. In the groups marked with an asterisk in the tables, one animal out of four was excluded due to technical issues.

Suppl table 5: Controls: 3 h after injection of monomers into the lateral ventricle or into the subarachnoïd space.
