# Supplementary material for: Transfer of human α-synuclein from the olfactory bulb to interconnected brain regions in mice
Source: Acta Neuropathol. 2013 Aug 8;126(4):555–73. doi: 10.1007/s00401-013-1160-3 (PMC3789892; doi:10.1007/s00401-013-1160-3)
Supplement: Supplementary file 2 — Supplementary Figure 1 (PDF 87 kb) [file 401_2013_1160_MOESM2_ESM.pdf]

Supplementary figure 1

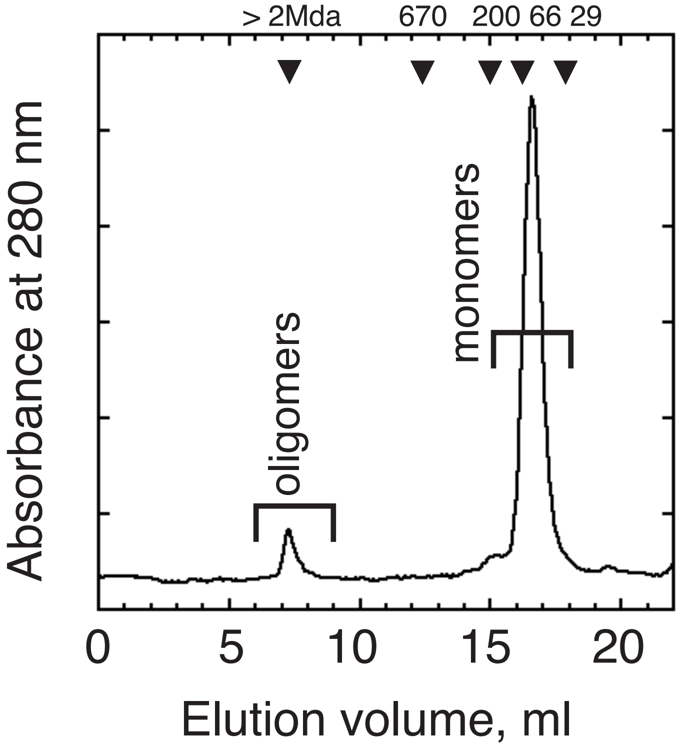

Supplementary figure 1. Separation of monomeric and oligomeric α-syn species by size exclusion chromatography (SEC).

Samples of 100 µL at 10 *µ*M of α-syn assemblies were loaded on a Superose 6 (10/300) sizing column (GE Healthcare Life Sciences) at 4°C and eluted isocratically in assembly buffer at a flow rate of 0.5 mL/min. Elution was monitored by UV absorbance at 280nm. The column was calibrated using Dextran blue (>2 MDa), thyroglobulin (670 kDa), ß-amylase (200 kDa), BSA (66 kDa), carbonic anhydrase (29 kDa). α-Syn oligomers are eluted in the first peak with apparent molecular weight of 2 MDa. Monomers are eluted at apparent molecular weight of 58 kDa. It is worth noting that SEC do not allow an accurate measurement of the size of the protein. Therefore, we cannot claim these molecular weight reflect the precise size of our proteins.
