# Supplementary material for: Transfer of human α-synuclein from the olfactory bulb to interconnected brain regions in mice
Source: Acta Neuropathol. 2013 Aug 8;126(4):555–73. doi: 10.1007/s00401-013-1160-3 (PMC3789892; doi:10.1007/s00401-013-1160-3)
Supplement: Supplementary file 3 — Supplementary Figure 2 (PDF 786 kb) [file 401_2013_1160_MOESM3_ESM.pdf]

Supplementary figure 2

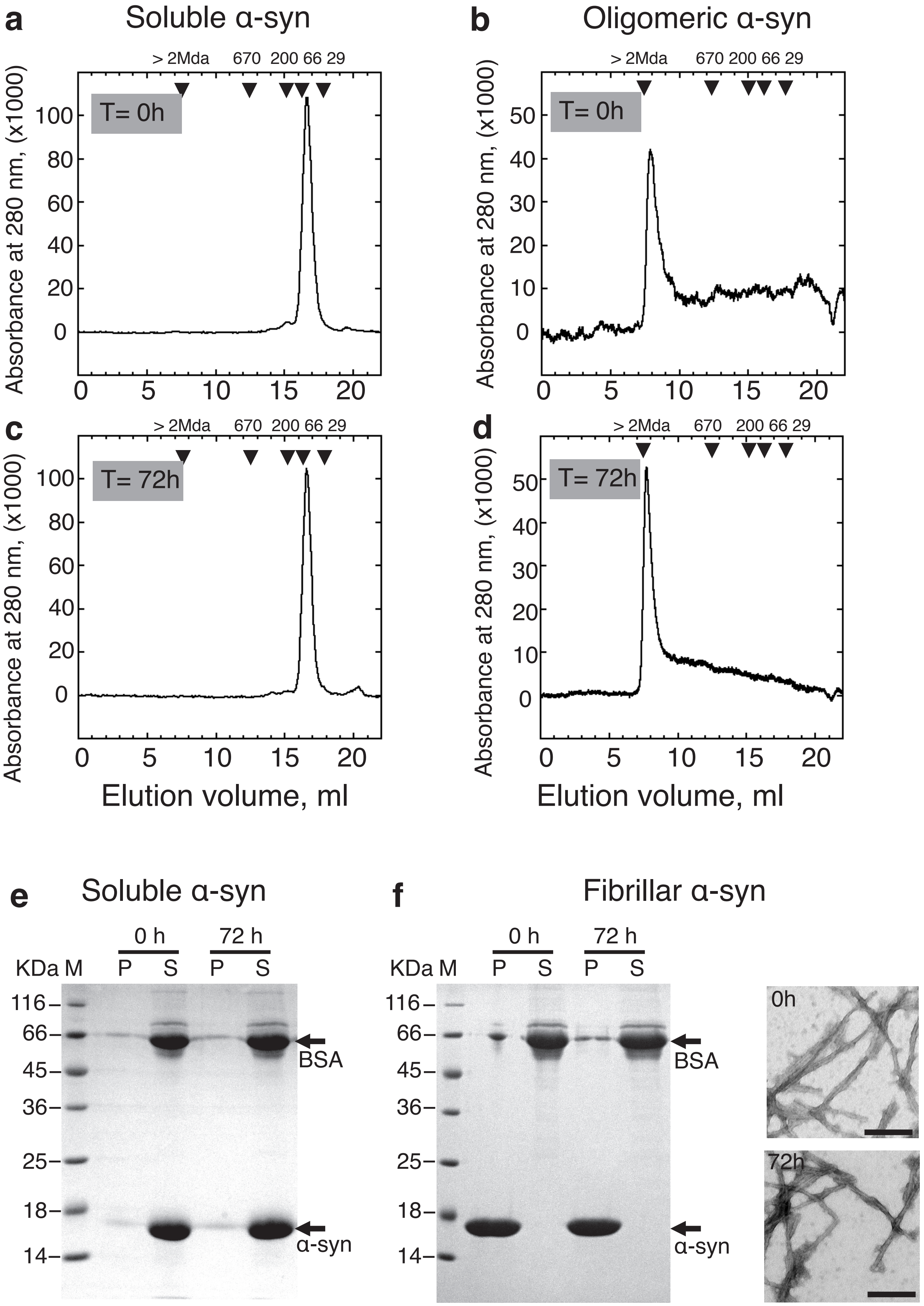

Supplementary figure 2: Stability of recombinant monomeric, oligomeric and fibrillar assemblies over 72h.

a-d. Stability of recombinant monomeric and oligomeric α-syn over 72 h duration in assembly buffer.

a. and **b.** Size exclusion chromatography (SEC) of soluble and oligomeric α-syn assemblies at time zero.

c. and **d.** SEC of soluble and oligomeric α-syn assemblies after 72 h in assembly buffer at 37°C, without shaking. The corresponding monomer and oligomer peaks were unchanged, revealing that these assemblies are stable over the time of the experiment.

e-f. Stability of recombinant monomeric and fibrillar α-syn assemblies (100 μM) over a 72 h incubation in culture media containing fetal bovine serum. Standard SDS-polyacrylamide gel electrophoresis was performed in 10% gels following the method described by Laemmli (Laemmli, U. K. (1970) Nature 227, 680–685). Gels were stained with coomassie blue.

e. Sedimentation assay (30.000 g, 18°C, 30 min) of soluble α-syn at initial (0 h) and after 72 h.

f. Sedimentation assay of α-syn fibrils at initial (0 h) and after 72 h. Soluble and fibrillar α-syn are not degraded over 72 h, nor polymerized for soluble α-syn or depolymerized for α-syn fibrils. Molecular weight marker (M), pellet (P), supernatant (S).
