# Supplementary material for: Transfer of human α-synuclein from the olfactory bulb to interconnected brain regions in mice
Source: Acta Neuropathol. 2013 Aug 8;126(4):555–73. doi: 10.1007/s00401-013-1160-3 (PMC3789892; doi:10.1007/s00401-013-1160-3)
Supplement: Supplementary file 4 — Supplementary Figure 3 (PDF 2186 kb) [file 401_2013_1160_MOESM4_ESM.pdf]

Supplementary figure 3

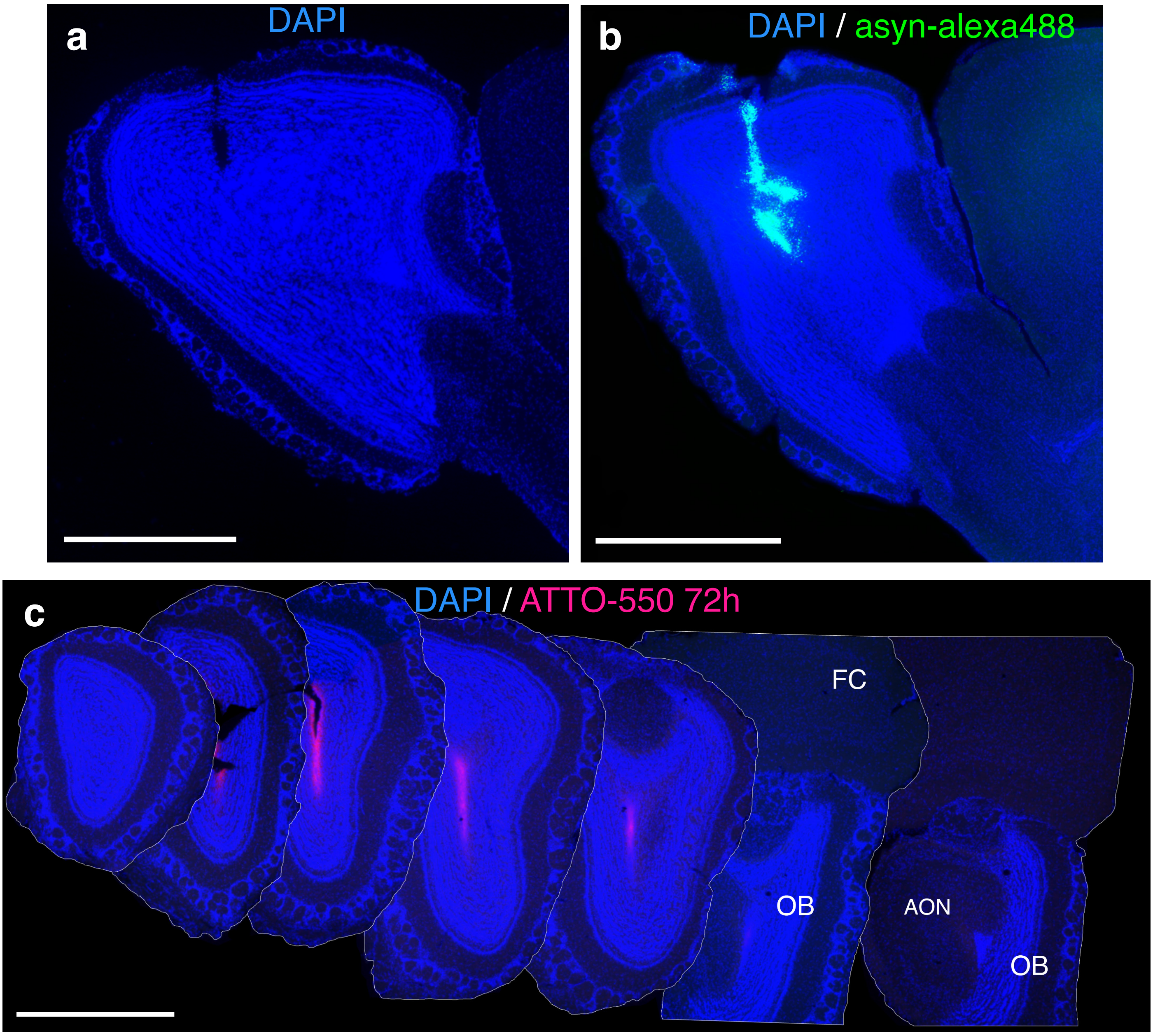

Supplementary figure 3: Stereotactic injections covered the rostrodorsal olfactory bulb with no significant diffusion to other structures up to 72h after injection.
a. Low magnification image showing the mechanical lesion in the olfactory bulb (OB) following intracerebral injection (DAPI labeling). **b.** Picture of OB injected with fibrillar asyn-alexa488 (green) after 12h (DAPI in blue) showing that the injection only spread into the rostrodorsal OB. The protein was taken up by some cells that were mechanically damaged during the injection, leading to an intense green signal along the injection track. **c.** We injected unbound ATTO-550 (in PBS) into the OB of mice to investigate the diffusion of the injected solution after 72 h. This panel is a montage of equally distributed coronal sections at different rostro-caudal levels of a brain injected with unbound ATTO-550 (inter-section interval = 450 µm). At 72 h after injection, ATTO-550 is present only in central parts of the OB, but not in lateral layers, and is absent in brain regions outside the OB. (FC: frontal cortex, AON: anterior olfactory nucleus). Scale bar = 1 mm.
