# Supplementary material for: Transfer of human α-synuclein from the olfactory bulb to interconnected brain regions in mice
Source: Acta Neuropathol. 2013 Aug 8;126(4):555–73. doi: 10.1007/s00401-013-1160-3 (PMC3789892; doi:10.1007/s00401-013-1160-3)
Supplement: Supplementary file 5 — Supplementary Figure 4 (PDF 668 kb) [file 401_2013_1160_MOESM5_ESM.pdf]

Supplementary figure 4

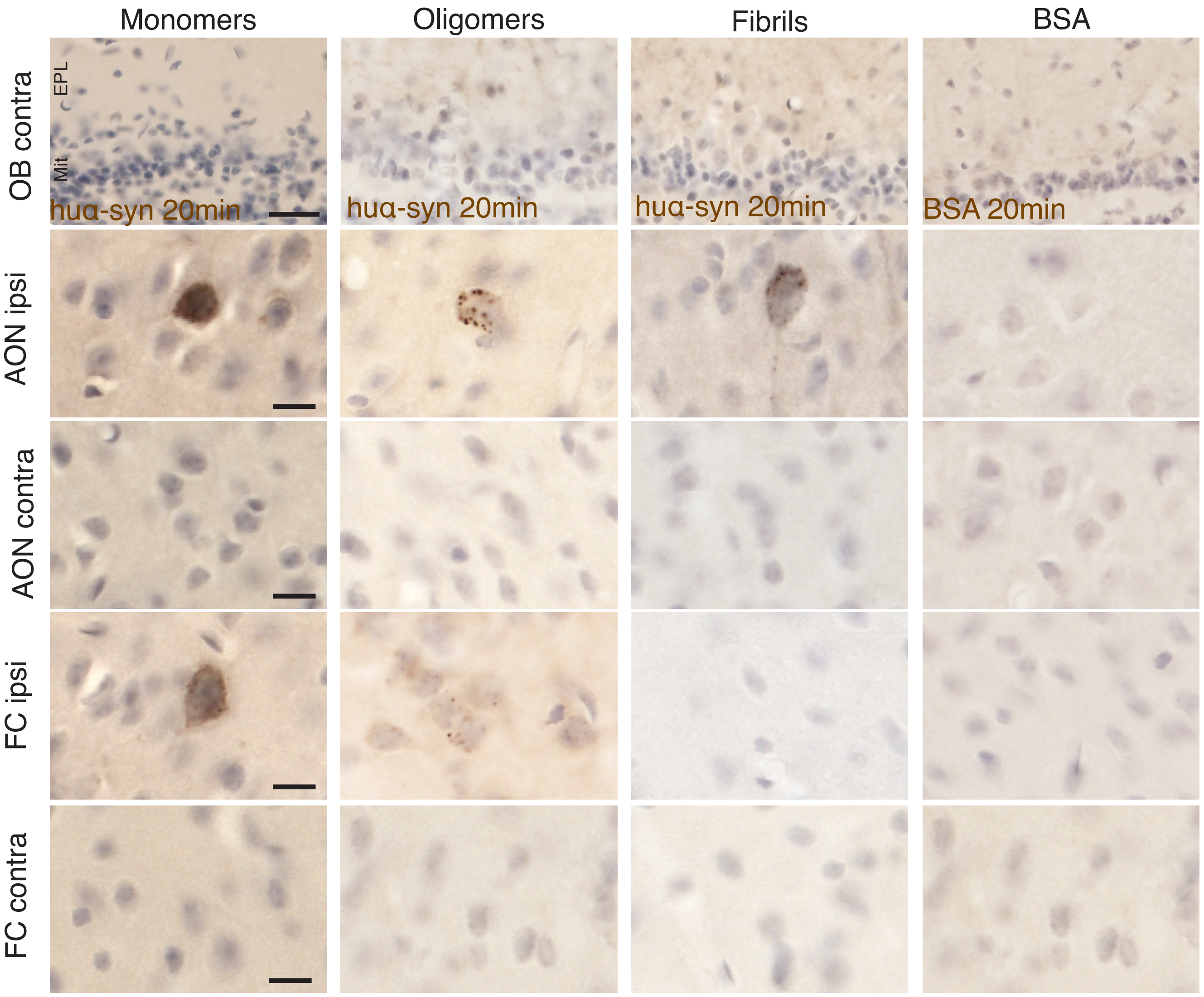


**Supplementary figure 4: Human α-synuclein or BSA staining in the anterior olfactory nucleus and frontal cortex 20 min after injection into the olfactory bulb.**
Low magnification images of OB (scale bar = 25 µm) and high magnification images of contralateral anterior olfactory nucleus (AON) and frontal cortex (FC) (scale bar = 10 µm), either ipsilateral (ipsi) or contralateral (contra) to the side of the injection. After injection of monomeric or oligomeric tα-syn, we identified huα-syn-positive cells in some mice in the ipsilateral AON, and rarely in FC. After injection of fibrillar tα-syn, we occasionally detected huα-syn-positive cells in some animals in the ipsilateral AON. When the injected protein was tBSA, we did not find BSA-positive cells in the brain. Mit: mitral layer; EPL: external plexiform layer.
