# Supplementary material for: Transfer of human α-synuclein from the olfactory bulb to interconnected brain regions in mice
Source: Acta Neuropathol. 2013 Aug 8;126(4):555–73. doi: 10.1007/s00401-013-1160-3 (PMC3789892; doi:10.1007/s00401-013-1160-3)
Supplement: Supplementary file 6 — Supplementary Figure 5 (PDF 2070 kb) [file 401_2013_1160_MOESM6_ESM.pdf]

Supplementary figure 5

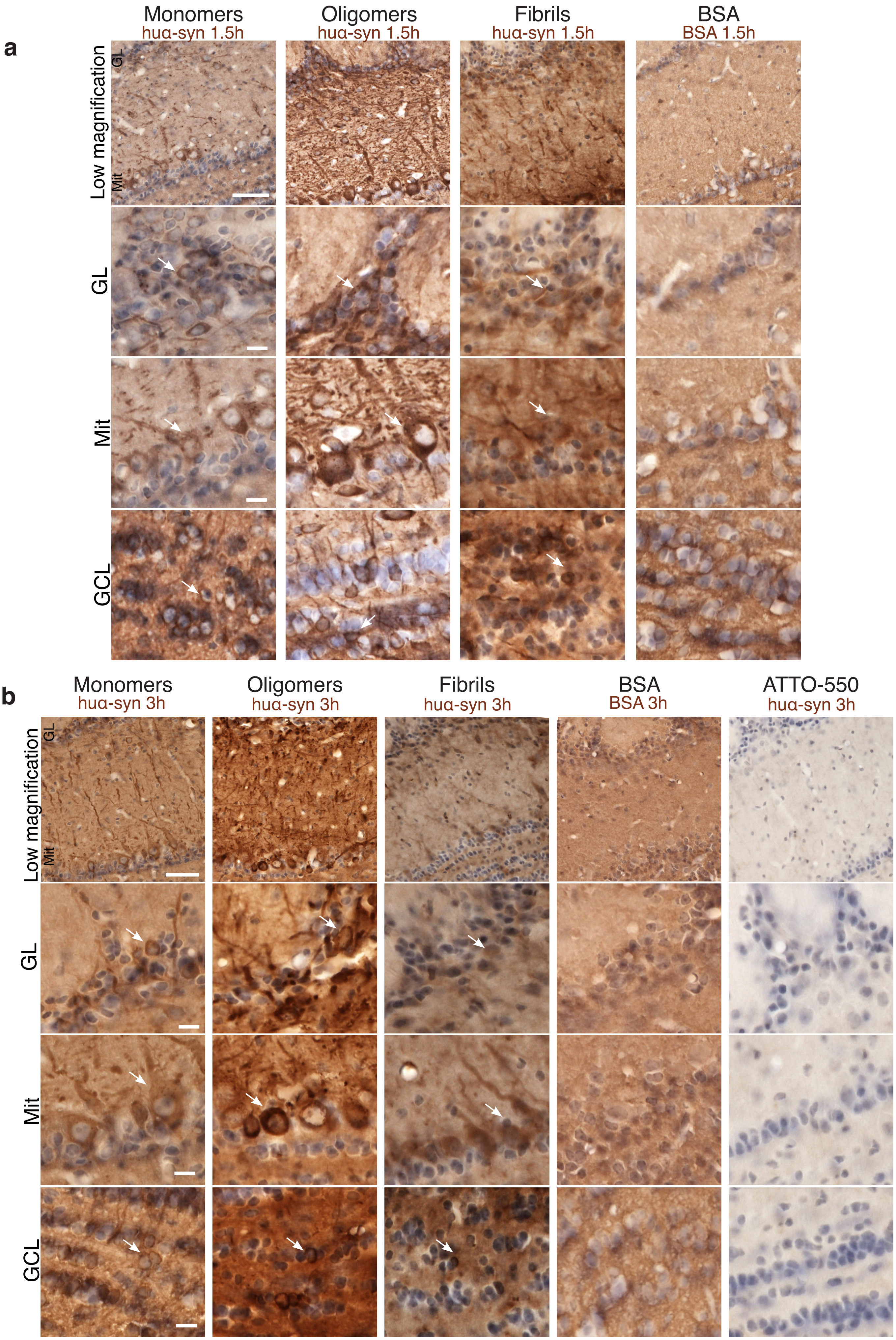

Supplementary figure 5: Human α-synuclein or BSA staining in the olfactory bulb 1.5 h and 3 h after protein injection into the olfactory bulb.

Low magnification images of the ipsilateral OB (Mit/ GL) (scale bar = 50 μm) or high magnification images (scale bar = 10 μm) of glomerular layer (GL), mitral layer (Mit), and granular cell layer (GCL) of the ipsilateral OB. **a.** At 1.5 h after injection of monomeric, oligomeric and fibrillar tα-syn, we observed huα-syn-positive cells in various layers of the OB, namely GL, Mit, and GCL. By contrast, no BSA-positive cell was found in the OB injected with tBSA. **b.** At 3 h after injection of monomeric, oligomeric and fibrillar tα-syn, we found huα-syn-positive cells still present in the GL, Mit, and GCL of the OB. By contrast, 3 h after injection of tBSA no BSA-positive cell was found in OB. As a control, we injected OB with ATTO-550 and stained with the syn211 antibody. In these mice we did not find any huα-syn-positive signal.
