# Supplementary material for: Transfer of human α-synuclein from the olfactory bulb to interconnected brain regions in mice
Source: Acta Neuropathol. 2013 Aug 8;126(4):555–73. doi: 10.1007/s00401-013-1160-3 (PMC3789892; doi:10.1007/s00401-013-1160-3)
Supplement: Supplementary file 7 — Supplementary Figure 6 (PDF 3961 kb) [file 401_2013_1160_MOESM7_ESM.pdf]

Supplementary figure 6- monomers

a

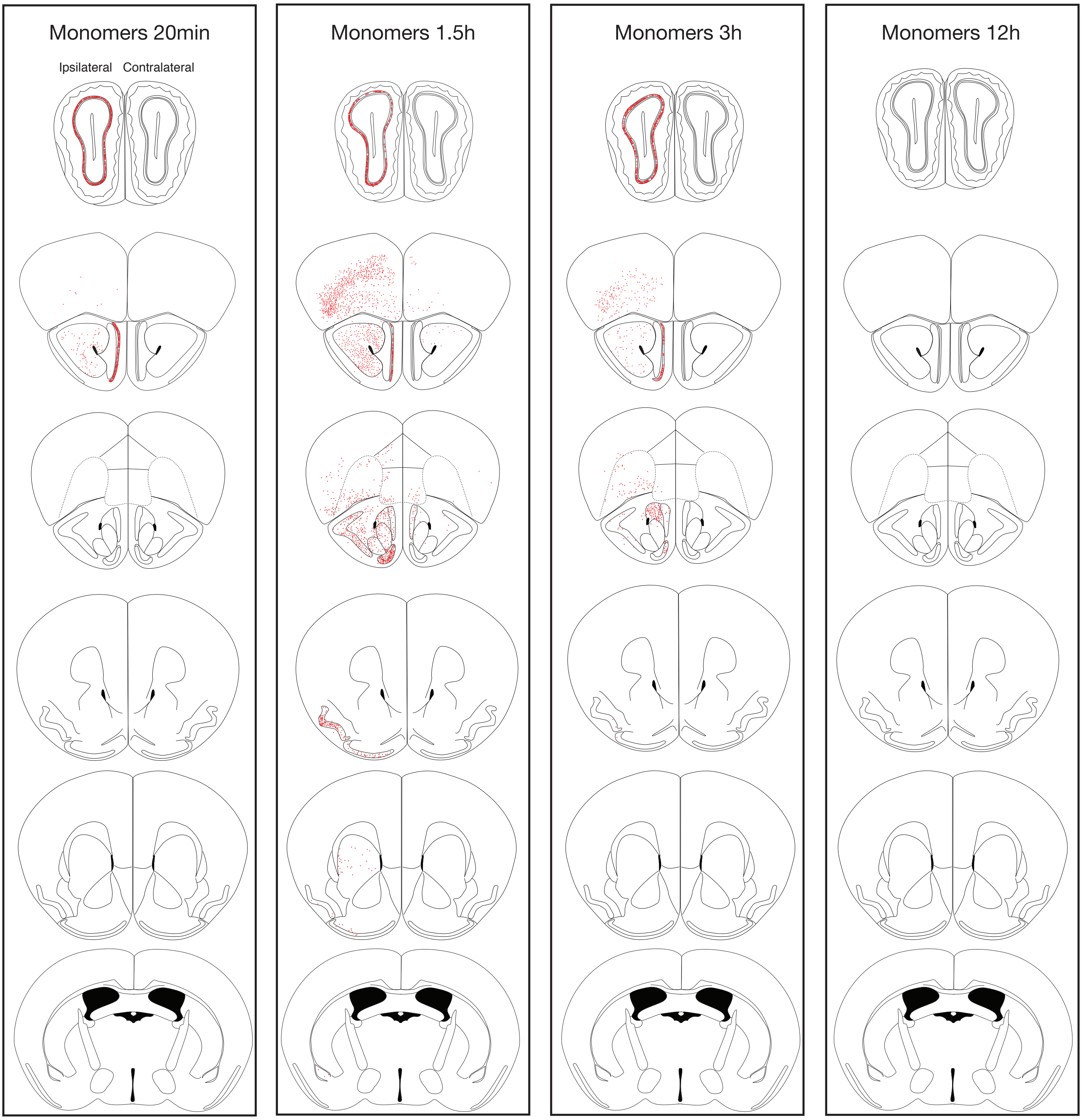

Supplementary figure 6a: Sketches representing the distribution of human-ɑ-synuclein-positive cells in the brains of mice injected with monomers

Based on brain maps we obtained from our cell quantification program (MBF stereoinvestigator), sketches based on mouse brain Paxinos atlas 2001 were drawn in Illustrator (Adobe), and location of positive cells was marked by red dots. For 20 min, 3h, and 12 h time points, one animal was mapped per group, taken randomly. For the 90 min time point, 4 animals per group were analysed, and mice presented here were the mice showing maximal transfer. In the olfactory bulb, only mitral cells were quantified, and are indicated here by red circles. To fully appreciate the pattern of cell labelling, it is necessary to zoom in on the high definition sketches.

Supplementary figure 6 - oligomers

b

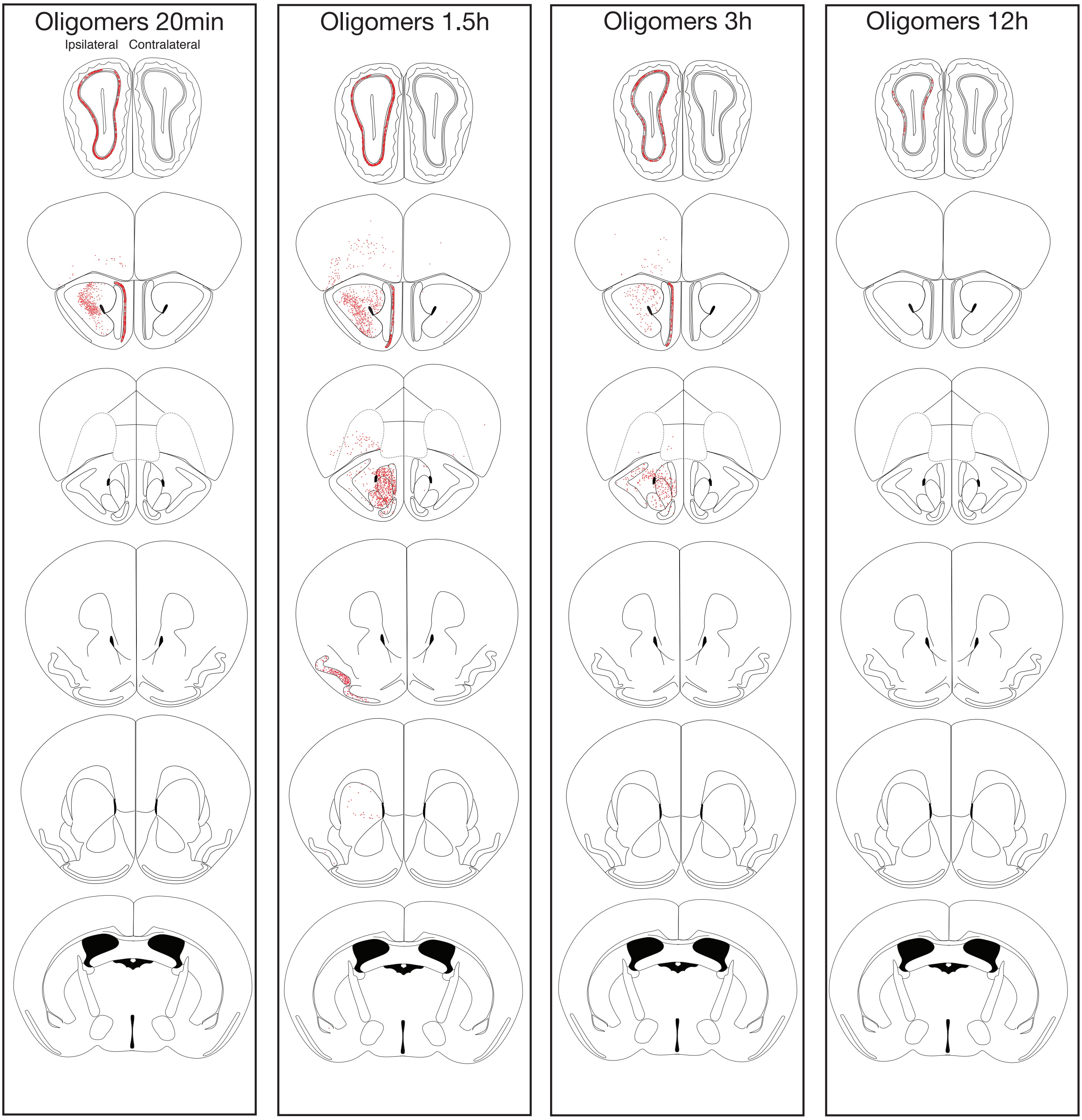

Supplementary figure 6b: Sketches representing the distribution human-α-synuclein-positive cells in the brains of mice at different time points following injection with oligomers.

Supplementary figure 6 - fibrils

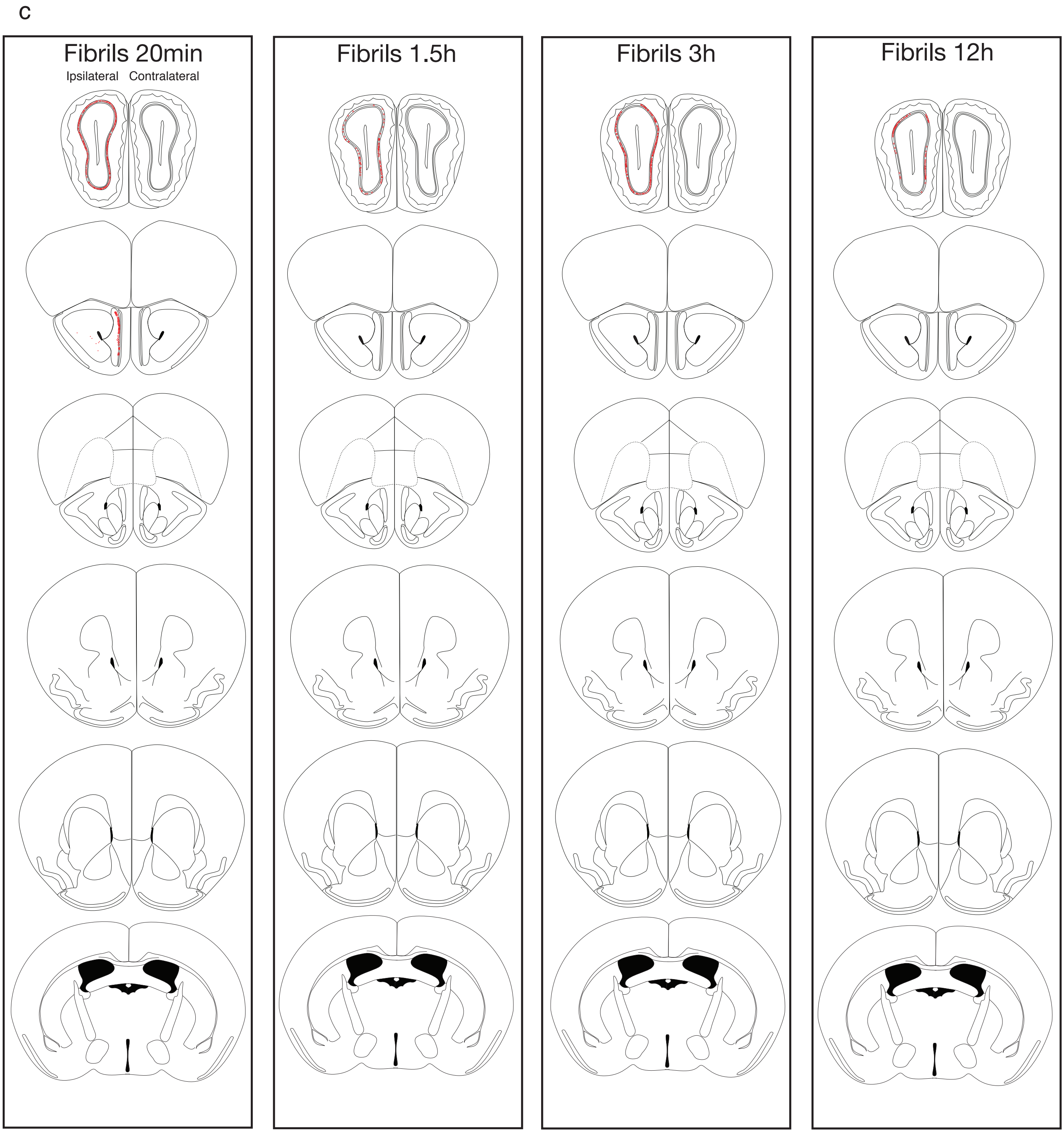

Supplementary figure 6c: Sketches representing the distribution of human-α-synuclein-positive cells in the brains of mice at different time points following injection with fibrils.
