# Supplementary material for: Transfer of human α-synuclein from the olfactory bulb to interconnected brain regions in mice
Source: Acta Neuropathol. 2013 Aug 8;126(4):555–73. doi: 10.1007/s00401-013-1160-3 (PMC3789892; doi:10.1007/s00401-013-1160-3)
Supplement: Supplementary file 8 — Supplementary Figure 7 (PDF 712 kb) [file 401_2013_1160_MOESM8_ESM.pdf]

Supplementary figure 7

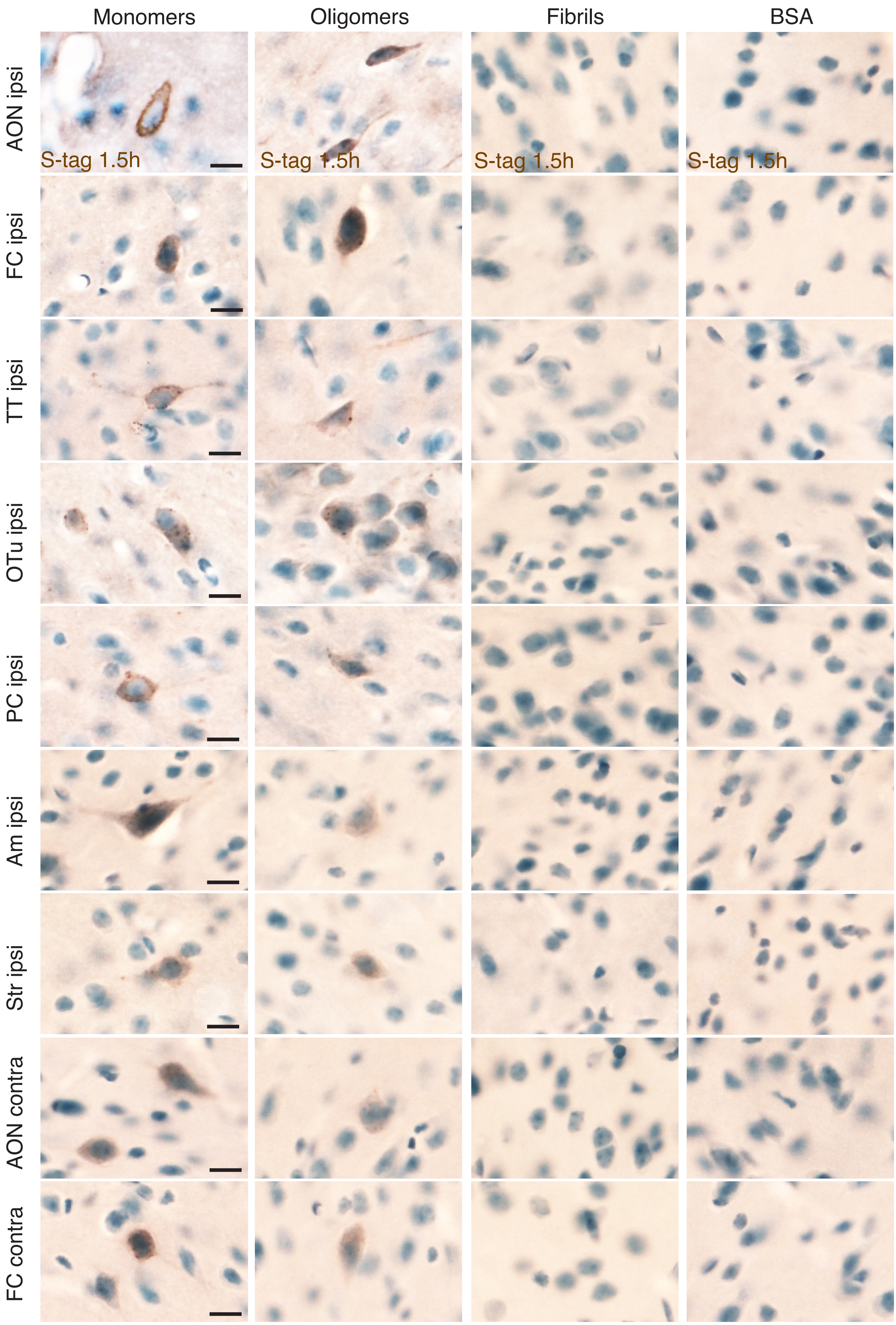

Supplementary figure 7: S-tag staining 1.5 h after injection of tagged α-synuclein or tBSA into the olfactory bulb.

Images of S-tag staining at high magnification (scale bar represents 10 µm) in various brain areas 1.5 h after injection of monomeric and oligomeric tα-syn into the OB. S-tag-positive cells were present in the ipsilateral and contralateral anterior olfactory nucleus (ipsi/ contra AON), in ipsi- and contralateral frontal cortex (ipsi/contra FC), in ipsilateral tenia tecta (ipsi TT), in ipsilateral olfactory tubercle (ipsi Otu), ipsilateral piriform cortex (ipsi PC), amygdala (ipsi Am) and striatum (ipsi Str). Moreover, S-tag-positive cells were observed only in the ipsilateral OB when we injected fibrillar tα-syn. tBSA injected as a control protein into the OB was not tagged with S-tag, and was not detected by anti-S-tag staining. Scale bar represents 10 µm.
