# Supplementary material for: Transfer of human α-synuclein from the olfactory bulb to interconnected brain regions in mice
Source: Acta Neuropathol. 2013 Aug 8;126(4):555–73. doi: 10.1007/s00401-013-1160-3 (PMC3789892; doi:10.1007/s00401-013-1160-3)
Supplement: Supplementary file 9 — Supplementary Figure 8 (PDF 376 kb) [file 401_2013_1160_MOESM9_ESM.pdf]

Suppl figure 8

Untagged Monomers

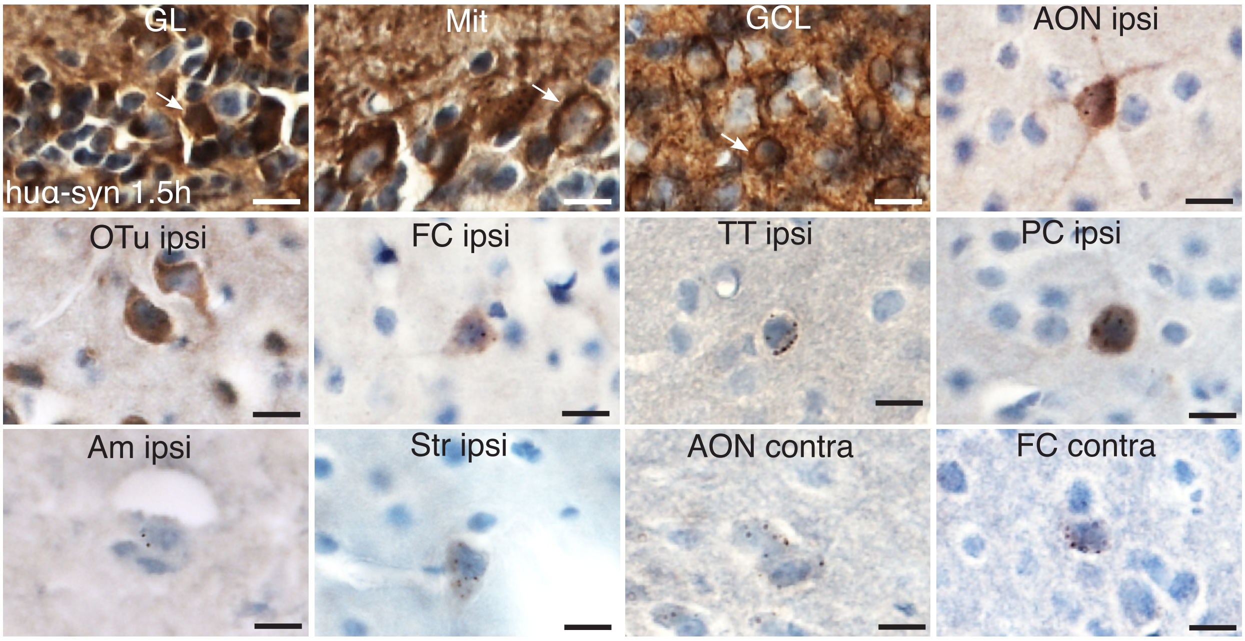

Supplementary figure 8: Human-α-synuclein staining 1.5 h after injection of untagged α-syn monomers into the OB.

Pictures of hu-α-syn staining at high magnification (scale bar represents 10 µm) in various brain areas 1.5 h after injection. Hu-α-syn-positive cells were detected in the same structures 1.5 h after the injection of monomeric untagged α-syn into the OB, as observed after injections of tagged α-syn. Hu-positive cells were present in the ipsi- and contralateral anterior olfactory nucleus (ipsi/ contra AON), in ipsi- and contralateral frontal cortex (ipsi/contra FC), in ipsilateral tenia tecta (ipsi TT), in ipsilateral olfactory tubercle (ipsi Otu), ipsilateral piriform cortex (ipsi PC), amygdala (ipsi Am) and striatum (ipsi Str). Scale bar represents 10 µm.
