# Supplementary material for: Transfer of human α-synuclein from the olfactory bulb to interconnected brain regions in mice
Source: Acta Neuropathol. 2013 Aug 8;126(4):555–73. doi: 10.1007/s00401-013-1160-3 (PMC3789892; doi:10.1007/s00401-013-1160-3)
Supplement: Supplementary file 10 — Supplementary Figure 9 (PDF 521 kb) [file 401_2013_1160_MOESM10_ESM.pdf]

Supplementary figure 9

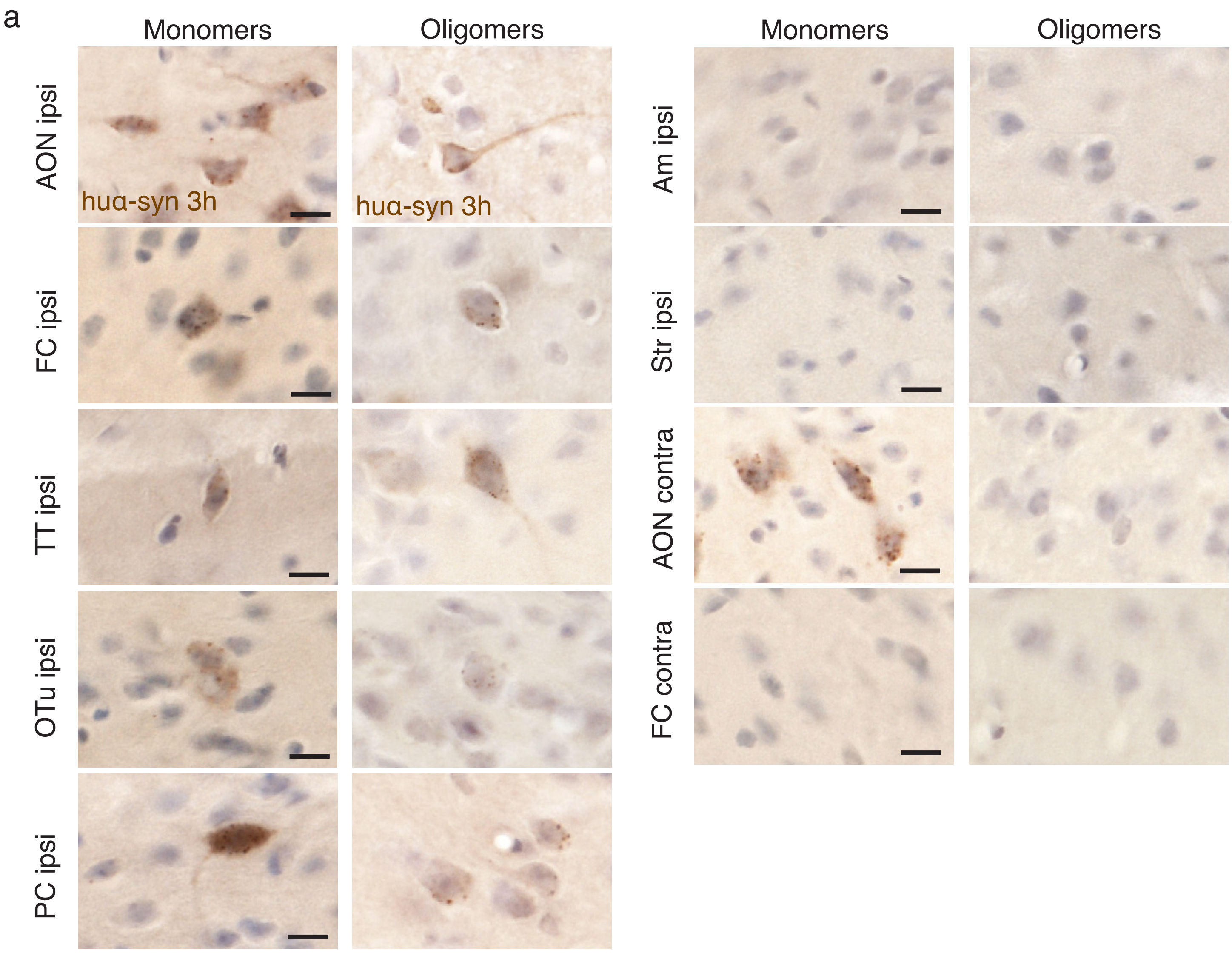

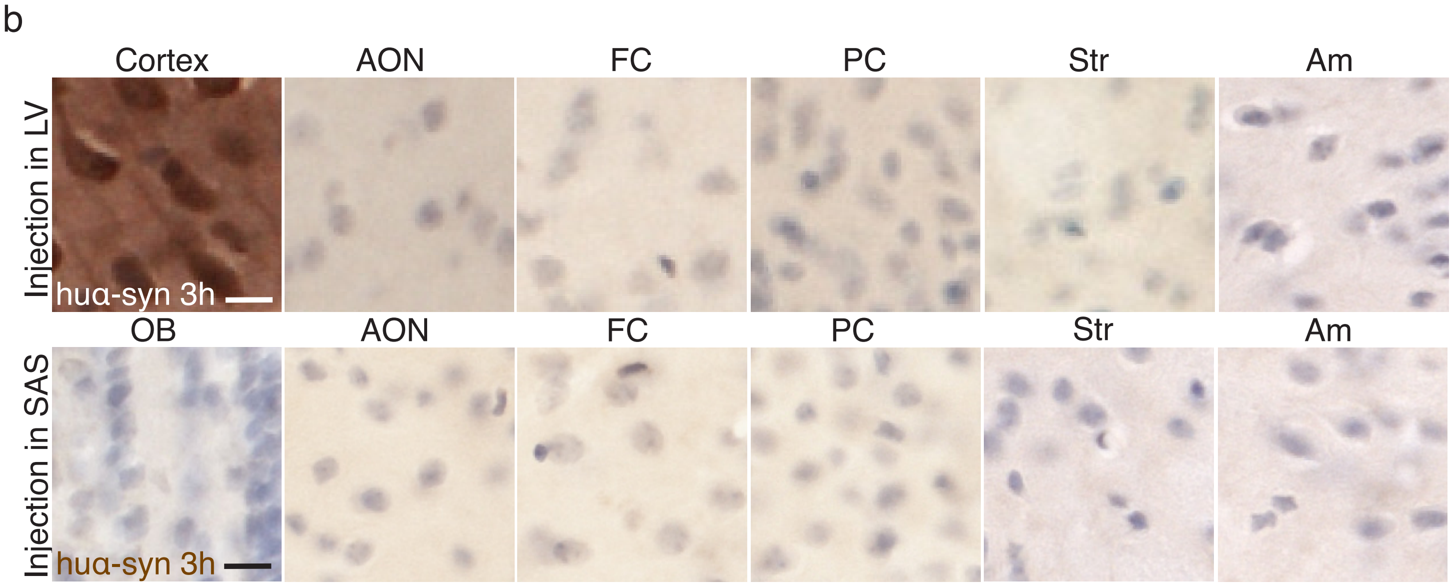

Supplementary figure 9: Huα-syn staining 3 h after injection into the OB, and control of diffusion through cerebrospinal fluid.

a. We observed huα-syn-positive cells in the bilateral AON, in ipsilateral FC, TT, OTu, and PC when monomeric tα-syn was injected. At 3 h after we injected oligomeric tα-syn, we found huα-syn-positive cells only in ipsilateral AON, FC, TT, OTu, and PC. When the injected tα-syn was fibrillar, no huα-syn-positive cell was detected in other structures than the OB (not shown here). Scale bar represents 10 µm.

b. Control of diffusion of tα-syn through cerebrospinal fluid. We injected two groups of mice with monomeric tα-syn into the lateral ventricle (LV) or in the subarachnoid space (SAS) on the top of the OB. We analysed these brains 3 h after injection to confirm that the presence of tα-syn in further structures could not be due to a diffusion of injected tα-syn through the cerebrospinal fluid. We stained the sections with an antibody (syn211) that recognize specifically human α-syn (huα-syn). We did not detect any huα-syn-positive cell in the brain, except in the cortex around the injection site when tα-syn was injected into the LV. Olfactory bulb (OB), anterior olfactory nucleus (AON), frontal cortex (FC), piriform cortex (PC), Striatum (Str), Amygdala (Am). Scale bar represents 10 µm.
